# Kernel-based genetic association analysis for microbiome phenotypes identifies host genetic drivers of beta-diversity

**DOI:** 10.1186/s40168-023-01530-0

**Published:** 2023-04-20

**Authors:** Hongjiao Liu, Wodan Ling, Xing Hua, Jee-Young Moon, Jessica S. Williams-Nguyen, Xiang Zhan, Anna M. Plantinga, Ni Zhao, Angela Zhang, Rob Knight, Qibin Qi, Robert D. Burk, Robert C. Kaplan, Michael C. Wu

**Affiliations:** 1 Department of Biostatistics, University of Washington, Seattle, WA 98195 USA; 2 Public Health Sciences Division, Fred Hutchinson Cancer Center, Seattle, WA 98109 USA; 3 Division of Biostatistics, Department of Population Health Sciences, Weill Cornell Medicine, New York, NY 10065 USA; 4 Department of Epidemiology and Population Health, Albert Einstein College of Medicine, Bronx, NY 10461 USA; 5 Institute for Research and Education to Advance Community Health, Washington State University, Seattle, WA 98101 USA; 6 Department of Biostatistics and Beijing International Center for Mathematical Research, Peking University, Beijing, 100191 China; 7 Department of Mathematics and Statistics, Williams College, Williamstown, MA 01267 USA; 8 Department of Biostatistics, Johns Hopkins University, Baltimore, MD 21205 USA; 9 Departments of Pediatrics, Computer Science & Engineering, and Bioengineering; Center for Microbiome Innovation, University of California, San Diego, La Jolla, CA 92093 USA; 10 Departments of Pediatrics; Microbiology & Immunology; and, Obstetrics, Gynecology & Women’s Health, Albert Einstein College of Medicine, Bronx, NY 10461 USA

**Keywords:** Microbiome, Beta-diversity, GWAS, Kernel machines, Covariate adjustment

## Abstract

**Background:**

Understanding human genetic influences on the gut microbiota helps elucidate the mechanisms by which genetics may influence health outcomes. Typical microbiome genome-wide association studies (GWAS) marginally assess the association between individual genetic variants and individual microbial taxa. We propose a novel approach, the covariate-adjusted kernel RV (KRV) framework, to map genetic variants associated with microbiome beta-diversity, which focuses on overall shifts in the microbiota. The KRV framework evaluates the association between genetics and microbes by comparing similarity in genetic profiles, based on groups of variants at the gene level, to similarity in microbiome profiles, based on the overall microbiome composition, across all pairs of individuals. By reducing the multiple-testing burden and capturing intrinsic structure within the genetic and microbiome data, the KRV framework has the potential of improving statistical power in microbiome GWAS.

**Results:**

We apply the covariate-adjusted KRV to the Hispanic Community Health Study/Study of Latinos (HCHS/SOL) in a two-stage (first gene-level, then variant-level) genome-wide association analysis for gut microbiome beta-diversity. We have identified an immunity-related gene, *IL23R*, reported in a previous microbiome genetic association study and discovered 3 other novel genes, 2 of which are involved in immune functions or autoimmune disorders. In addition, simulation studies show that the covariate-adjusted KRV has a greater power than other microbiome GWAS methods that rely on univariate microbiome phenotypes across a range of scenarios.

**Conclusions:**

Our findings highlight the value of the covariate-adjusted KRV as a powerful microbiome GWAS approach and support an important role of immunity-related genes in shaping the gut microbiome composition.

Video Abstract

**Supplementary Information:**

The online version contains supplementary material available at 10.1186/s40168-023-01530-0.

## Introduction

The human microbiome plays an important role in host health and is involved in fundamental body functions such as metabolism and immune response [13, 47]. While environmental factors have a large influence on microbiome composition [52], it is still of interest to study the effect of human genetic variation on the microbiome: such studies not only help us understand the hereditary component of the human microbiome, but also provide clues as to the biological mechanisms by which genetics may influence health outcomes. As a notable example, elevated abundance of *Bifidobacterium*, a genus of beneficial gut bacteria that utilizes lactose as an energy source, has been associated with a non-persistence genotype of the human lactase gene (*LCT*), which typically results in lactose intolerance [6, 26, 38]. Such an association implies a potential mediating role of the gut microbiome in the relationship between host genetics and metabolic outcomes, where the presence of Bifidobacteria may provide some level of lactose tolerance to lactase non-persistent individuals [26].

Many studies have sought to identify genetic variants that influence microbial composition, and most of them incorporate microbiome characteristics as phenotypes in genome-wide association studies (GWAS). Typical analyses marginally test the association between abundances of individual taxa and genotypes of individual genetic variants [8, 17, 33, 38]. Such analyses often suffer from a low statistical power, due to a large multiple-testing burden and failure to accommodate inherent structure in microbiome and genetic data, e.g., phylogenetic relationships among taxa and epistasis among genetic variants.

As the microbiome functions as a community, an alternative microbiome phenotype is beta-diversity, the dissimilarity in overall microbiome profiles between individuals. Beta-diversity analysis represents a standard mode of analysis in microbiome profiling studies as it focuses on discovery of concerted shifts in the community rather than individual taxa. However, few studies have considered beta-diversity as a trait of interest in microbiome GWAS and there is no standard strategy. Some studies [6, 62] have performed principal coordinates analysis (PCoA) on the pairwise beta-diversity matrix and evaluated the association between the top principal coordinates (PCos) and the genotype of each genetic variant. Such a strategy could suffer from power loss, as the top PCos may not fully capture the variation within the microbiome data. Hua et al. [32] assumed a linear model between the pairwise beta-diversity and the pairwise genetic distance at each genetic variant and developed a score test called microbiomeGWAS. Rühlemann et al. [53] adopted a distance-based multivariate analysis of variance (MANOVA) approach called distance-based F test [48] and evaluated the difference in beta-diversity among the different genotype groups for each genetic variant. These approaches still test one variant at a time and are subject to a stringent genome-wide significance threshold. Studies using the above approaches have identified loci within genes involved in immunity [6, 53], vitamin metabolism [62] and complex diseases such as type 2 diabetes [43]. In our study, we aim to further improve statistical power with a novel approach and bring more discoveries from microbiome GWAS.

Here, we propose to assess the association between groups of variants at the gene level and the overall microbiome composition, characterized by beta-diversity, at the community level. Community-level analyses and multi-variant testing have been shown to be powerful in microbiome [51, 70] and genetic studies [63], respectively, due to their ability to capture innate structure and correlation within the data, while reducing the multiple-testing burden. Using the recently developed kernel RV (KRV) framework [68, 69], we summarize individuals’ microbiome (or genetic) characteristics by a pairwise similarity matrix called “kernel” matrix, where each entry in the matrix represents similarity in microbiome (or genetic) profiles between a pair of individuals. Microbiome similarity can be obtained by transforming known beta-diversity measures, while genetic similarity can also be characterized in various ways, such as the average genotype matching over all genetic variants. The association between microbes and genetics is then assessed via comparing similarity in microbiome profiles to similarity in genetic profiles across all pairs of individuals. Intuitively, if the genetics is associated with the microbiome, we would expect the pairwise microbial profiles to be similar whenever the pairwise genetic profiles are similar. In particular, the test statistic is the normalized Frobenius inner product, a measure of correlation, between the two kernel matrices.

Although the KRV is a potentially powerful approach for microbiome GWAS, the KRV framework lacks a general strategy to control for covariates such as population structure, which is imperative for any genetic association analysis. Here we extend the original KRV framework to allow for flexible covariate adjustment.

We apply the covariate-adjusted KRV to the Hispanic Community Health Study/Study of Latinos (HCHS/SOL) [35, 60] via a two-stage (first gene-level, then variant-level) genome-wide association analysis for gut microbiome. This is the first study to investigate the genetic effect on the overall gut microbiome composition, characterized by beta-diversity, in Hispanic/Latino populations. We have identified a gene (*IL23R*) reported in a previous microbiome genetic association study and discovered other novel genes related to immune functions. Furthermore, we have identified individual genetic variants and specific microbial taxa involved in these gene-microbiome associations. In addition, our simulation results show that the covariate-adjusted KRV maintains valid type I error rates in the presence of confounding and has a much greater power than other single-trait-based competing methods across a range of scenarios. Together, our proposed approach demonstrates good statistical properties and provides a powerful way to study the effect of human genetic variation on microbiome composition.

## Methods

### Overview of covariate-adjusted KRV

**Fig. 1:**
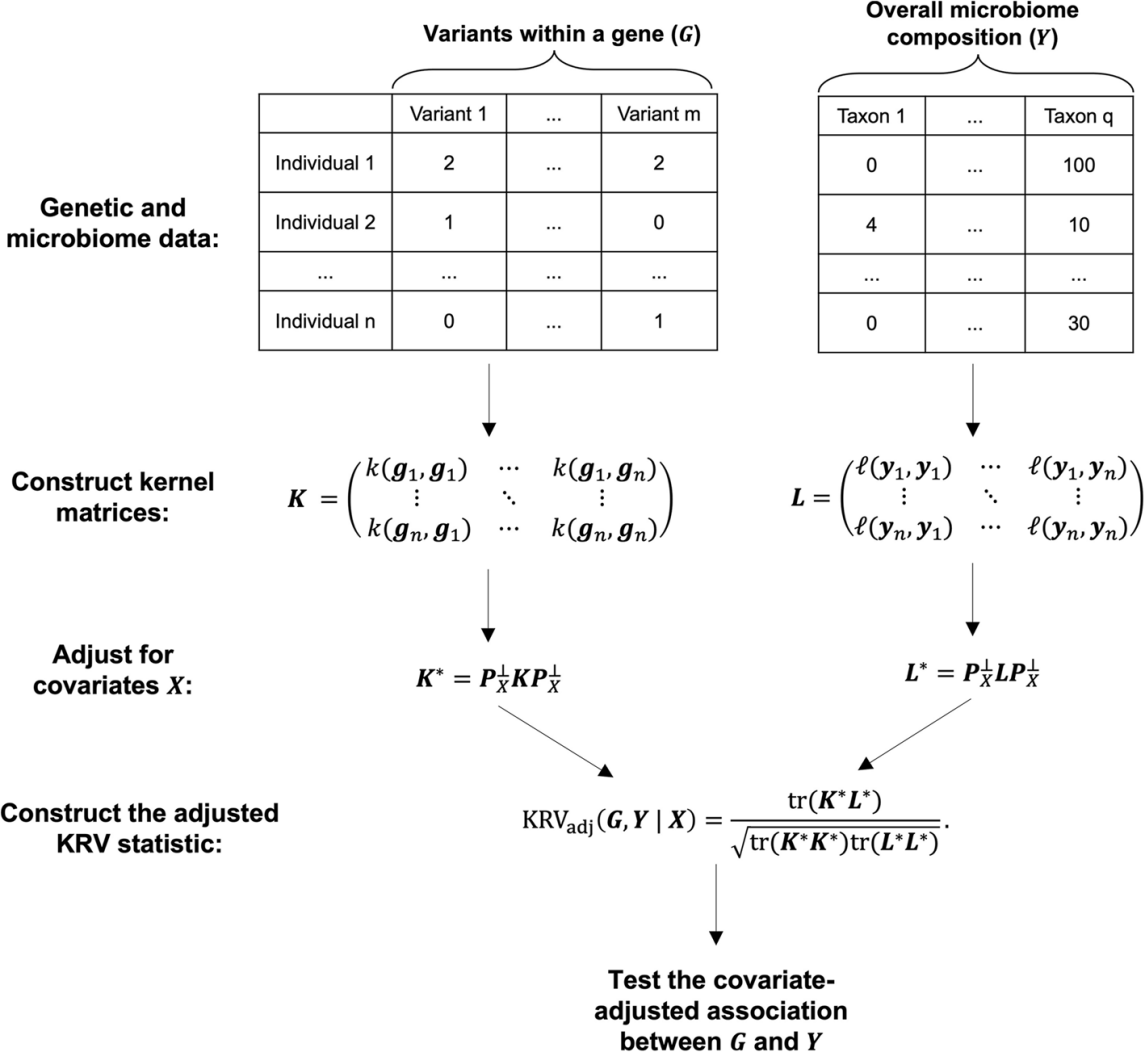
Illustration of covariate-adjusted KRV for microbiome genome-wide association studies

We aim to assess the covariate-adjusted association between genotypes of multiple genetic variants within a gene and abundances of microbial taxa at the community level, using the previously developed KRV framework. We now give an overview of the original KRV framework and extend it to allow for flexible covariate adjustment. The overall procedure for covariate-adjusted KRV in the context of microbiome GWAS is shown in Fig. 1.

The KRV framework has been proposed by Zhan et al. [68, 69] to evaluate the general association between a group of genetic variants, *G*, and a group of traits, *Y*. Suppose we have genotype data of *m* genetic variants and phenotype data of *q* traits available for *n* unrelated individuals. For the *i-*th subject, let $\varvec{g}_i = (g_{i1}, \cdots , g_{im})^T$ be the set of genotypes, where $g_{il} \in \{0, 1, 2\}$ represents the number of minor alleles for the *l-*th variant; let $\varvec{y}_i = (y_{i1}, \cdots , y_{iq})^T$ be the set of traits. Example phenotypes in previous studies include expression values of multiple genes from a particular pathway [69] and levels of multiple amino acids [19]. In the context of microbiome GWAS, we treat the microbiome as the phenotype. Specifically, $\varvec{g}_i$ represents the genotypes of *m* genetic variants within a particular gene, and $\varvec{y}_i$ represents the abundances of *q* microbial taxa that form the microbiota.

Let $k(\varvec{g}_i, \varvec{g}_j)$ be a kernel function that measures the similarity in genetic profiles between individuals *i* and *j*. Let $\ell (\varvec{y}_i, \varvec{y}_j)$ be another kernel function that measures the similarity in phenotypic profiles between *i* and *j*. Specific choices of kernel functions in the context of microbiome GWAS are discussed in Methods: 2.2. We can then define a kernel matrix $\varvec{K} \in \mathbb {R}^{n \times n}$, where the (*i*, *j*)-th entry of $\varvec{K}$ is $k(\varvec{g}_i, \varvec{g}_j)$. Similarly, we define another kernel matrix $\varvec{L} \in \mathbb {R}^{n \times n}$ such that $\varvec{L}_{ij} := \ell (\varvec{y}_i, \varvec{y}_j)$. The matrices $\varvec{K}$ and $\varvec{L}$ can be viewed as pairwise similarity matrices for genotypes and phenotypes, respectively. We further center the two kernel matrices: let $\tilde{\varvec{K}} := \varvec{H} \varvec{K} \varvec{H}$ and $\tilde{\varvec{L}} := \varvec{H} \varvec{L} \varvec{H}$, where $\varvec{H} = \varvec{I} - \textbf{11}^T /n$ is a column-centering matrix. Then the KRV coefficient that evaluates the relationship between the genetic variants and the traits is defined as

1 $${\mathrm{KRV}}\left(G,Y\right)\;:=\frac{\mathrm{tr}\left(\tilde{\varvec{K}}\tilde{\varvec{L}}\right)}{\sqrt{\mathrm{tr}\left(\tilde{\varvec{K}}\tilde{\varvec{K}}\right)\mathrm{tr}\left(\tilde{\varvec{L}}\tilde{\varvec{L}}\right)}}.$$

Intuitively, the KRV coefficient compares genotypic similarity to phenotypic similarity across all pairs of individuals. A large KRV coefficient indicates that the pairwise similarity pattern in genetic profiles well resembles the pairwise similarity pattern in phenotypic profiles, which implies that the genetic variants are associated with the traits in a certain way. To perform hypothesis testing, the permutation distribution of the KRV statistic under the null hypothesis of no association between genetics and phenotypes can be approximated by a Pearson Type III distribution [69], allowing us to obtain a *p*-value and assess the significance of the association at a given significance level.

The above framework does not take into account any covariates that might be involved in a typical genetic association study. Now suppose that, for each individual *i*, we have a set of covariates $\varvec{x}_i = (1, x_{i1}, \cdots , x_{ip})^T \in \mathbb {R}^{p+1}$; let $\varvec{X} \in \mathbb {R}^{n \times (p+1)}$ be the sample covariates matrix such that the *i*-th row of $\varvec{X}$ is $\varvec{x}_i^T$. Assume that $\varvec{X}$ has full rank. We intend to assess the association between the genetic variants and the phentoypes, after adjusting for the effects of covariates $\varvec{X}$. Previous studies, including the original KRV framework, have suggested using a residual-based approach [9, 63, 69], where we first regress out the covariates from each raw phenotype and then construct the phenotype kernel matrix using the resulting residuals. Such an approach is not universally feasible for all microbiome kernels, as certain popular microbiome kernels (e.g., the Bray-Curtis kernel and the unweighted UniFrac kernel) require the input to be discrete taxa count data or taxa presence/absence data, which is not satisfied by the covariate-adjusted residuals. Furthermore, adjustment based on linear regression may not account for the potentially nonlinear relationships between the genetics/microbiome and the covariates.

To adjust for covariates in a general way, we propose a novel adjustment approach that applies to all possible kernel types, regardless of the requirement for input data. Our approach is based on kernel principal component analysis (kernel PCA) [55], a general and nonlinear extension of regular PCA, of the kernel matrices. Specifically, we first perform a kernel PCA on the constructed phenotype kernel matrix and treat the resulting kernel PCs as surrogate phenotypes, which could capture both linear and nonlinear features of the original phenotype data depending on the kernel function used. We then regress out the covariates from all kernel PCs and reconstruct the phenotype kernel matrix with the adjusted PCs. By adjusting the covariates on all kernel PCs, we are able to fully account for the variation within the phenotype data. The same procedure is performed on the genotype kernel matrix. After algebraic manipulation (see Additional File 1: Section S1), the adjusted KRV coefficient is of the form:

$$\begin{aligned} \text {KRV}_{adj} (G, Y | X) := \frac{\textrm{tr}( \varvec{K}^{*} \varvec{L}^{*})}{\sqrt{ \textrm{tr}(\varvec{K}^{*} \varvec{K}^{*} ) \textrm{tr}(\varvec{L}^{*} \varvec{L}^{*} ) }}, \end{aligned}$$

where $\varvec{K}^{*}:= \varvec{P}_{X}^\perp \varvec{K} \varvec{P}_{X}^\perp$, $\varvec{L}^{*}:= \varvec{P}_{X}^\perp \varvec{L} \varvec{P}_{X}^\perp$, $\varvec{P}_{X}^\perp := \varvec{I} - \varvec{P}_{X}$ and $\varvec{P}_{X}$ is the projection matrix onto the column space of $\varvec{X}$. We adjust for covariates on both the phenotype kernel and the genotype kernel, due to the symmetry of the KRV coefficient. Our proposed approach for covariate adjustment is able to capture both linear and nonlinear relationships between the genetics/microbiome and the covariates, and thus can be viewed as a general extension of the previous residual-based approach. When a linear kernel is used, our strategy is exactly equivalent to the residual-based approach (see Additional File 1: Section S1).

The usual hypothesis testing procedure in the KRV framework can be applied to the adjusted KRV statistic to obtain a *p*-value. In this case, the null hypothesis is that there is no association between the genetics and the phenotypes after adjusting for the effects of the covariates.

### Choice of kernels

In the KRV framework, kernel functions are used to summarize pairwise similarities in genotype and phenotype profiles among the subjects. In order to improve the statistical power in hypothesis testing, we would like to choose kernels that better reflect the actual structure within the genetic and phenotype data as well as the patterns of association [22, 70]. For the KRV statistic in (1) to be well-defined theoretically, the kernel matrices need to be positive semi-definite. We now review some of the common kernels used for genetic and microbiome data.

For genotype data, popular kernel functions include the linear kernel $k(\varvec{g}_i, \varvec{g}_j) = \varvec{g}_i^T \varvec{g}_j$ and the identity-by-state (IBS) kernel $k(\varvec{g}_i, \varvec{g}_j) = \frac{1}{2m} \sum _{l=1}^m (2 - |g_{il} - g_{jl}|)$. The linear kernel assumes that the genetic variants are associated with the traits in a linear fashion. The IBS kernel defines pairwise similarity as the pairwise genotype matching averaged over all genetic variants, and is useful when there are epistatic effects among the variants [63]. Depending on analysis interests (e.g. rare-variant analysis), it is also possible to incorporate a weight for each variant in the linear and IBS kernels [63].

For microbiome data at the community level, the kernel matrix can be obtained by transforming known ecological or phylogenetic dissimilarity measures (i.e., beta-diversity measures). For example, Bray-Curtis dissimilarity quantifies the dissimilarity between two microbial communities based on the difference in counts at each taxon between the two communities. The UniFrac distances are dissimilarity measures based on the phylogenetic structure of the taxa [11, 45, 46]: the unweighted UniFrac distance is calculated as the fraction of branch lengths within the phylogenetic tree that are not shared between the two communities; the weighted UniFrac distance further incorporates taxa abundance information on the basis of the unweighted distance; the generalized UniFrac distance is a compromise between weighted and unweighted UniFrac distances.

While the Bray-Curtis dissimilarity and UniFrac distances take scaled or rarefied microbial counts or presence/absence information as input, microbial dissimilarity can also be calculated from other types of transformed abundance data. For example, the centered log-ratio (CLR) transformation [2, 24] and phylogenetic isometric log-ratio (PhILR) transformation [57] have been proposed to address the compositional nature of microbiome data, where PhILR further incorporates phylogenetic information into the transformed data. As these log-ratio-based transformations encourage normality, Euclidean distances can then be calculated based on the CLR-transformed or PhILR-transformed data as measures of dissimilarity.

Given a pairwise dissimilarity matrix $\varvec{D}$, the corresponding kernel matrix can be constructed as:

$$\begin{aligned} \varvec{L} = -\frac{1}{2} \left( \varvec{I} - \frac{\textbf{11}^T}{n} \right) \varvec{D}^2 \left( \varvec{I} - \frac{\textbf{11}^T}{n} \right) , \end{aligned}$$

where $\varvec{D}^2$ is the element-wise square of $\varvec{D}$. To ensure that the kernel matrix $\varvec{L}$ is positive semi-definite, we further apply a correction procedure as implemented in the MiRKAT R package [70], where we perform an eigendecomposition of $\varvec{L}$, convert any negative eigenvalues to zero and then reconstruct the kernel matrix.

We note that taking Euclidean distances followed by kernel matrix transformation is equivalent to constructing a linear kernel matrix based on the same data (see Additional File 1: Section S1). Therefore, the kernels derived from Euclidean distances of CLR- and PhILR-transformed data can be viewed as linear kernels directly applied to these transformed data. We denote the resulting kernel matrices as CLR-linear and PhILR-linear kernels, respectively.

### Description of the HCHS/SOL study

Hispanic Community Health Study/Study of Latinos (HCHS/SOL) is a community-based prospective cohort study aimed to identify risk factors for health outcomes in Hispanic/Latino populations in the USA. The study recruited 16,415 Hispanic/Latino adults aged 18–74 years, representing diverse ethnic background, at four US field centers (Bronx, NY, Chicago, IL, Miami, FL, and San Diego, CA), using a two-stage probability sampling design [60].

12,803 participants consented to genetic studies. Genotyping was performed on an Illumina custom array, SOL HCHS Custom 15041502 B3, which consisted of the Illumina Omni 2.5M array (HumanOmni2.5-8v1-1) and $\sim$150,000 custom SNPs [15]. Quality control, genotype imputation and estimation of pairwise kinship coefficients and PCs of genome-wide genetic variability were described in detail by Conomos et al. [15]. In addition to the quality control procedures described in [15], prior to the microbiome GWAS analysis, we also filtered imputed genetic variants based on an “effective minor allele count”: $N_{\text {eff}} = 2 \hat{p} (1- \hat{p})N v$, where $\hat{p}$ is the estimated minor allele frequency, *N* is the sample size and *v* is the ratio of observed variance of imputed dosages to the expected binomial variance [41]. We retained variants with sufficient minor allele counts and excluded any variants with $N_{\text {eff}} < 30$.

As an ancillary study, the HCHS/SOL Gut Origins of Latino Diabetes (GOLD) study was further conducted to investigate the role of gut microbiome composition in diabetes and other health outcomes in Hispanic/Latino individuals [35]. Gut microbiome profiles were available in 1674 participants, a subset of the HCHS/SOL participants. Based on the collected stool samples, DNA extraction and 16S rRNA gene sequencing were performed according to the Earth Microbiome Project (EMP) standard protocols [23]. Subsequent bioinformatic processing of the microbiome sequencing data was described in detail by Kaplan et al. [35].

The HCHS/SOL study was approved by the Institutional Review Boards of all participating institutions, and written informed consent was obtained from all participants.

### Microbiome GWAS analysis of HCHS/SOL data

To identify genetic variants associated with the overall gut microbiome composition in Hispanic/Latino individuals, we applied the covariate-adjusted KRV test to the HCHS/SOL study in a genome-wide association analysis for gut microbiome beta-diversity.

We considered genetic variants (including both single-nucleotide polymorphisms, or SNPs, and insertion/deletion variants, or indels) within $\pm 10$ kb of gene regions along Chromosomes 1–22 and grouped the variants into gene-level variant-sets correspondingly. The microbiome operational taxonomic units (OTUs) were collapsed at the genus level. We used a linear kernel for the genetic data and six different kernels for the microbiome data, including Bray-Curtis, unweighted UniFrac, weighted UniFrac, generalized UniFrac, CLR-linear and PhILR-linear, as described in Methods: 2.2. Rarefied microbial abundance data were used to construct Bray-Curtis and UniFrac kernels, while absolute abundance data were used to construct CLR-linear and PhILR-linear kernels, where a unit pseudo-count was added to address zero entries before CLR and PhILR transformations. The weightings used in PhILR transformation were the same as those proposed in [57].

For each gene, we assessed the association between common variants (with minor allele frequency, or MAF, $\ge$ 0.05) within the gene and the community-level microbiome profile, using both adjusted and unadjusted KRV tests. In the adjusted KRV, we mainly controlled for the top 5 PCs of genome-wide genetic variability (denoted as the PC-adjusted KRV), as they were shown to well capture the population structure of the sample based on a previous genetic study of HCHS/SOL data [15]. Individuals from different populations and ethnic groups often have systematic differences in their genetic and microbiome profiles [16, 65], so population structure is an important confounder in our analysis. We also performed additional analyses that adjusted for other non-confounding covariates including age, gender and study sites.

To avoid confusion, we emphasize the distinction between (1) kernel PCs derived from the kernel matrices, as mentioned in Methods: 2.1 and (2) genome-wide genetic PCs. In the context of our gene-level microbiome GWAS, the kernel PCs of the genotype kernel matrix capture information of a particular gene that we are interested in testing against the microbiome. On the other hand, the genome-wide genetic PCs capture genetic information along the entire genome and are used as covariates to measure population structure. In the PC-adjusted KRV analysis, the top 5 genome-wide genetic PCs were regressed out from all kernel PCs of the gene-level genotype kernel matrix and all kernel PCs of the community-level microbiome kernel matrix.

Our investigation of the genetic effect on the microbiome involved two stages. In the first stage, we tested the association between the variants in each gene and the microbiome profile at the community level. In the second stage, for any genes called significant in the first stage, we marginally assessed the association between each of the individual variants within those genes and the community-level microbiome profile to look for significant variants, again using the covariate-adjusted KRV. Bonferroni correction was applied in both stages. Since this was a nested hypothesis testing approach, the second-stage test only required correction for the number of variants in the genes that were called significant in the first stage. All analyses were performed on unrelated individuals (pairwise kinship coefficient $\le 0.05$) where genetic data, microbiome data and covariates data were available.

As a comparison to our proposed covariate-adjusted KRV approach, we applied additional microbiome GWAS approaches to the same sample. First, we considered two methods that still analyze the association between gene-level genetic variation and community-level microbiome composition but use univariate approaches. One method was linear regression, where we performed kernel PCA on both the gene-level genotype kernel matrix and the community-level microbiome kernel matrix and regressed the top kernel PC of the microbiome kernel on the top kernel PC of the genotype kernel, while adjusting for covariates. The other method was SNP-set kernel association test (SKAT) [63], a kernel machine regression framework for assessing the general association between a univariate trait and multiple genetic variants. Here we performed kernel PCA on the community-level microbiome kernel matrix and used the SKAT test to regress the top kernel PC of the microbiome kernel on the genetic variants within each gene, while adjusting for covariates; a linear kernel was used for genetic data in the SKAT test. In addition to gene-based community-level competing methods, we also conducted a traditional variant-based taxon-level microbiome GWAS, where we tested the association between individual genetic variants along the genome and individual microbial genera present in $\ge 10\%$ of all participants. A detailed analysis procedure for the taxon-level analysis is described in Additional File 1: Section S2. In all the competing methods, the top 5 PCs of genome-wide genetic variability were adjusted as covariates.

### Simulation studies

We conducted simulation studies to further evaluate the type I error rate and power of the covariate-adjusted KRV test. We simulated genotype data and microbial OTU count data under realistic settings, and introduced population stratification as a confounder that affected both genetic and microbiome data.

The general simulation setting is as following. We considered a sample size of 1000. SNP genotype data over a 1-Mb chromosome were simulated for 500 individuals of African ancestry and 500 individuals of European ancestry. Specifically, we first generated 10,000 haplotypes of African ancestry and another 10,000 haplotypes of European ancestry over a 1-Mb chromosome according to coalescent theory using the *cosi2* program [56]. To form a sample, we then generated the genotype of each African individual in the sample by randomly selecting and pairing 2 haplotypes from the 10,000 founding African haplotypes. A similar procedure was used to generate the genotypes of European individuals.

We used a Dirichlet-multinomial distribution to generate microbial OTU counts for each individual in the sample, as this distribution well accommodates the over-dispersion of microbiome count data [12, 70]. To ensure a realistic simulation of OTU counts, we estimated the parameters of the Dirichlet-multinomial distribution from a real upper-respiratory-tract microbiome data set [10], which consisted of 856 OTUs. This data set is publicly available as part of the GUniFrac R package. We assumed 1000 total OTU counts per individual. Population structure was introduced into the OTU count data in two ways, as described below.

Both unadjusted and adjusted KRV tests were performed to test the association between the overall microbiome composition (composed of 856 OTUs) and common SNPs (with MAF $\ge$ 0.05) within an 8-kb subregion of the 1-Mb chromosome. This 8-kb subregion can be considered as a simulated gene region. In the adjusted KRV test, the top PC of genetic variability (obtained from PCA on SNP data over the entire 1-Mb region) was used as the covariate, a surrogate for population structure. We considered a linear kernel for genetic data and six different kernels for microbiome data: Bray-Curtis, unweighted UniFrac, weighted UniFrac, generalized UniFrac, CLR-linear and PhILR-linear.

To evaluate type I error rates in the presence of confounding, we introduced population structure into the OTU count data in two scenarios (denoted as Type I Error Scenario 1 and 2). In Type I Error Scenario 1, we increased the abundance of the 10 most common OTUs by 10% in African individuals and then rarefied the abundance back to 1000 total counts per individual. In Type I Error Scenario 2, we increased the abundance of 10 rare OTUs (chosen randomly from the top 40 rarest OTUs) in African individuals by adding a unit count before rarefying the abundance back to 1000 total counts per individual. These two scenarios were not meant to reflect the microbiome difference between African and European individuals in reality, but they served as hypothetical situations to introduce confounding effect into the genetics-microbiome relationship. Here we used the estimated mean proportion parameters of the Dirichlet-multinomial distribution as a measure of OTU prevalence. 10,000 simulations were performed for each type I error scenario.

To evaluate the power of the covariate-adjusted KRV, we based our simulation setting on Type I Error Scenario 1 and further introduced genetic effect on the microbiome in three different power scenarios, where a single SNP affected the abundance of multiple microbial OTUs (i.e., a pleiotropy effect). Let $g_i$ be the genotype (0, 1 or 2) of individual *i* at a chosen common SNP. In Power Scenario 1, for each individual *i*, we increased the counts of the 11th–20th most common OTUs by a factor of $f_i$, where $f_i=1+c_1 g_i$. In Power Scenario 2, utilizing the available phylogenetic tree for the 856 OTUs [10], we increased the counts of OTUs from a relatively abundant cluster (representing 10.3% abundance of the total OTU counts) by a factor of $f_i$ for each individual *i*, where $f_i=1+c_2 g_i$. In Power Scenario 3, for each individual *i*, we increased the counts of 5 rare OTUs (chosen randomly from the top 40 rarest OTUs) by an addition of $a_i$, where $a_i = c_3 g_i$. We considered two sets of effect sizes: (a) small effect sizes: $c_1 = c_2 = 0.3, c_3 = 0.5$ and (b) large effect sizes: $c_1 = 0.8, c_2=0.7, c_3 = 1$. After introducing these genetic effects on the microbiome, we again rarefied the OTU counts to 1000 total counts per individual. For each power scenario, 1000 simulations were performed.

In the power simulation, we also considered two competing methods that analyze the association between a group of variants and the overall microbiome composition but rely on univariate microbiome phenotypes, as described in Methods: 2.4. The first method was linear regression, where we regressed the top kernel PC of the community-level microbiome kernel matrix on the top kernel PC of the gene-level genotype kernel matrix, while adjusting for covariates. The second method was SKAT, where we applied the SKAT test to regress the top kernel PC of the microbiome kernel on the genetic variants within the pre-specified gene region, while adjusting for covariates; we used a linear kernel for genetic data in the SKAT test.

### Computation time

We estimated the computation time of the covariate-adjusted KRV test for different sample sizes. For each sample size, we simulated 10 data sets and reported the average computation time. Given constructed genotype and microbiome kernel matrices and 10 covariates, the average computation times are 0.09, 1.23, 12.58, and 97.57 s on a laptop (2.7 GHz CPU and 16 GB memory) for sample sizes of 200, 500, 1000, and 2000, respectively. The gene-level analysis of the HCHS/SOL data set (with one genotype kernel, 6 microbiome kernels and 19,223 variant-sets) took approximately 8 hours on a high-performance computing cluster (each node with 24 cores, 3.00 GHz CPU and 384 GB memory), with computing jobs divided by chromosome.

## Results

### Application of covariate-adjusted KRV to HCHS/SOL

**Fig. 2:**
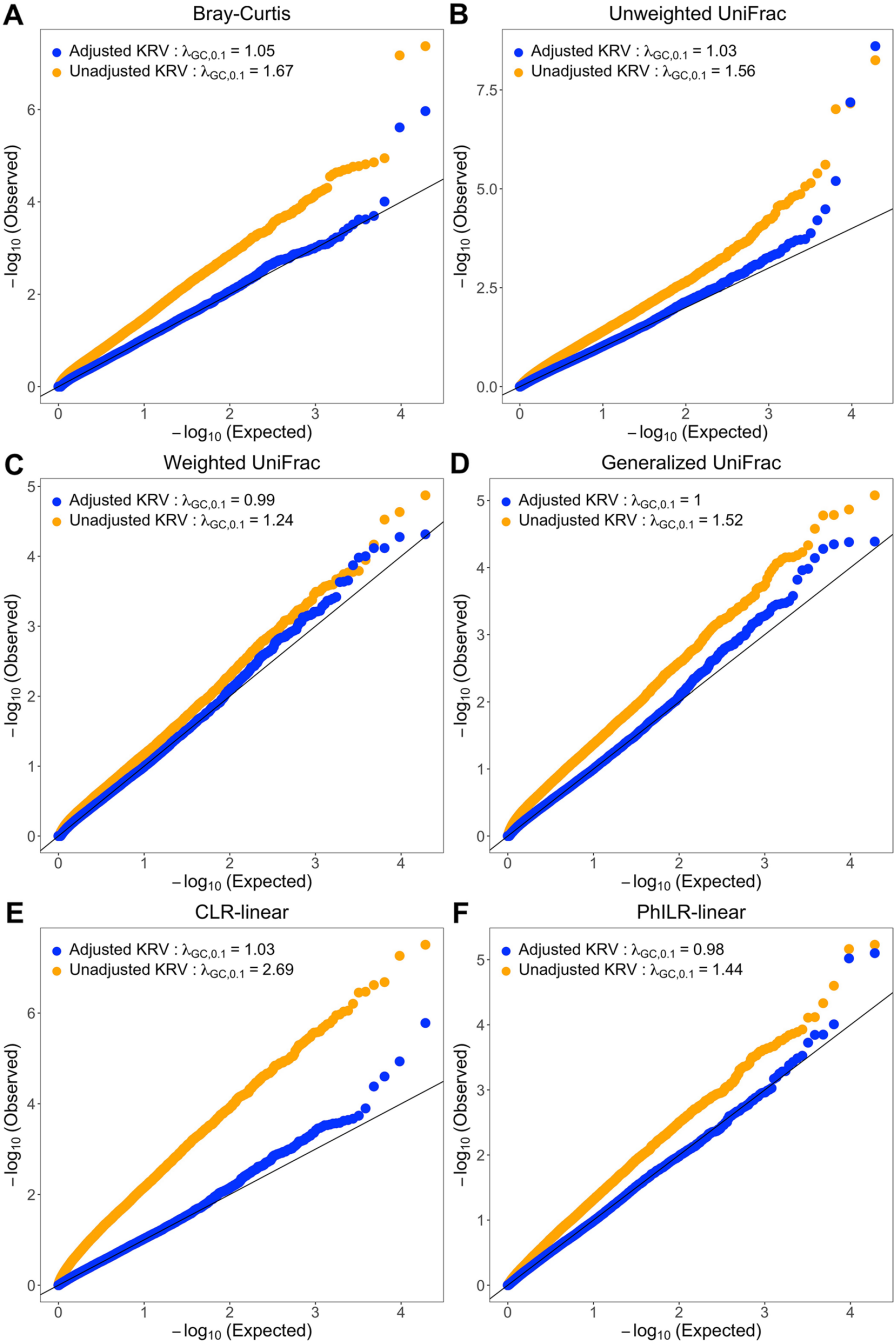
*P*-value QQ-plots from the first-stage gene-level analysis of the HCHS/SOL data. Each panel corresponds to a QQ-plot based on a distinct microbiome kernel. In the adjusted KRV, the top 5 PCs of genome-wide genetic variability were adjusted. $\lambda _{GC,0.1}$ represents the genomic inflation factor evaluated at the upper 10th percentile

We performed our microbiome GWAS analyses on 1219 unrelated participants from HCHS/SOL where all relevant data were available. Among these individuals, 47.0% identified their background as Mexican, 14.8% as Cuban, 12.7% as Puerto Rican, 10.3% as Central American, 7.7% as South American and 7.5% as Dominican. Microbiome count data were obtained on 408 genera, rarefied to 10,000 total counts per individual to construct Bray-Curtis and UniFrac kernels. A total of 19,223 gene-level variant-sets that contained at least one common variant were available. Figure 2 shows the *p*-value QQ-plots of the first-stage gene-level analysis results. For all microbiome kernels, the unadjusted KRV produces highly anti-conservative *p*-values (with large genomic inflation factors), while the PC-adjusted KRV has well-controlled type I error rates (with genomic inflation factors $\le 1.05$), confirming that population structure is the major confounder in our study. The gene-level Manhattan plots based on the PC-adjusted KRV are shown in Fig. S1.

Table 1 shows the genes identified at a genome-wide significance in the PC-adjusted first-stage analysis ($\alpha = 0.05/19,223=2.6 \times 10^{-6}$). We have found two genes, *IL23R* and *C1orf141*, using the Bray-Curtis kernel and two genes, *MTMR12* and *ZFR*, using the unweighted UniFrac kernel. *MTMR12* is also identified by the CLR-linear kernel. When the analysis is performed on a reduced set of individuals (*n*=1096) where additional covariates (age, gender, and study sites) are available and adjusted, *IL23R* and *C1orf141* are no longer genome-widely significant (Table S1). Similar non-significant results are observed for *IL23R* and *C1orf141* when only genome-wide genetic PCs are adjusted in the same subsample. To investigate the reason for this power loss, we perform PC-adjusted analyses on random subsamples of the same size from the original 1219 individuals. Around half of the times, at least two out of the four genes no longer have genome-wide significance, indicating that the non-significant results in the reduced sample are likely due to sample size loss, rather than systematic differences between the reduced sample and the original sample. Nevertheless, the results from the two adjusted analyses are similar in both their observed KRV statistics (*IL23R*: 0.017 in the original sample vs. 0.016 in the reduced sample; *C1orf141*: 0.018 in the original sample vs. 0.016 in the reduced sample) and the order of magnitude of their *p*-values ($10^{-6}$ in the original sample vs. $10^{-5}$ in the reduced sample). Additional analyses to assess the robustness of these two signals are reported in Additional File 1: Section S3.

**Table 1:** Significant genes identified from the first-stage gene-level analysis of the HCHS/SOL data, using the PC-adjusted KRV ($\alpha = 2.6 \times 10^{-6}$)

Microbiome kernel	Significant genes	Number of common variants	*P*-value
Bray-Curtis	*C1orf141*	484	$1.1 \times 10^{-6}$
	*IL23R*	284	$2.4 \times 10^{-6}$
Unweighted UniFrac	*MTMR12*	174	$6.5 \times 10^{-8}$
	*ZFR*	288	$2.5 \times 10^{-9}$
CLR-linear	*MTMR12*	174	$1.7 \times 10^{-6}$

The top 5 PCs of genome-wide genetic variability were adjusted

Among these genes, *IL23R* is of considerable interest: it encodes one part of the receptor for interleukin-23 (IL-23), a pro-inflammatory cytokine closely involved in autoimmunity [20]. The *IL23R* gene has been associated with inflammatory bowel diseases (IBD) including Crohn’s disease and ulcerative colitis [18, 58]. In a previous genetic association study of microbiome composition [67], the protective variant of the *IL23R* gene (rs11209026) was associated with a higher microbiome diversity and richness and a higher abundance of beneficial gut bacteria in the ileum of healthy individuals, suggesting the influence of host genetics on the microbiome prior to onset of IBD. In addition, a mouse-based experimental study [1] showed that mice deficient in intestinal *IL23R* expression had altered gut microbiota and were susceptible to colonic inflammation, where increased disturbance of gut microbiota exacerbated the disease activity. Coupled with these results, our finding further supports that the gut microbiome may mediate the host genetic effect on the development of inflammatory diseases like IBD. In its normal function, the *IL23R* gene likely helps shape the overall gut microbiota towards a healthy composition, which may in turn support normal immune activities and prevent gut inflammation.

The other genes are also interesting to further explore. The *C1orf141* gene, with uncharacterized protein function, has overlapping regions with *IL23R*. Variants in the *IL23R-C1orf141* region have been associated with susceptibility to Vogt-Koyanagi-Harada disease, a multi-system autoimmune disorder that affects pigmented tissues, in Chinese and Japanese populations [31, 54]. The *ZFR* gene encodes the highly conserved zinc finger RNA-binding protein, which is shown to prevent excessive type I interferon activation by regulating alternative pre-mRNA splicing [30]. Prevention of excessive type I interferon activation is important for the regulation of immune responses. The *MTMR12* gene encodes an adapter protein for myotubularin-related phosphatases and is likely involved in skeletal muscle functions [29]. Overall, most of the significant genes have a role in immunity, indicating an interaction between the host genetics and the gut microbiome in facilitating immune responses or developing autoimmune disorders.

**Fig. 3:**
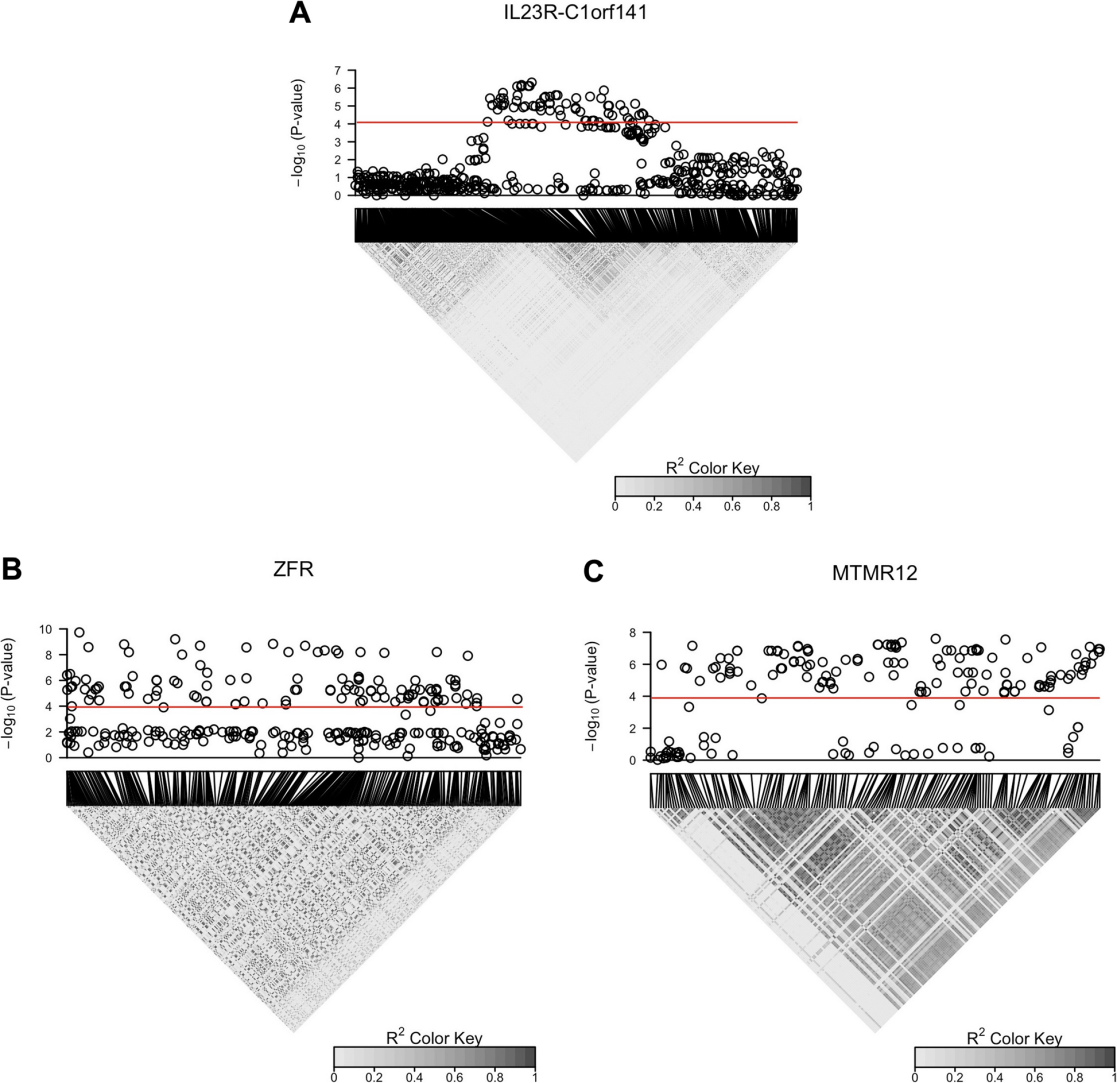
Manhattan plots and linkage disequilibrium (LD; $R^2$) heatmaps from the second-stage variant-level analysis of the HCHS/SOL data, using the PC-adjusted KRV. Each panel corresponds to a distinct gene or gene region. The Bray-Curtis kernel was used for analysis of variants in the *IL23R-C1orf141* region; the unweighted UniFrac kernel was used for analysis of variants in *ZFR* and *MTMR12*. The top 5 PCs of genome-wide genetic variability were adjusted. The red lines represent variant-level significance after Bonferroni correction ($\alpha = 8.98 \times 10^{-5}$ for variants in the *IL23R-C1orf141* region, and $1.08 \times 10^{-4}$ for variants in *ZFR* and *MTMR12*). A large $R^2$ value indicates high LD

As *MTMR12* is more significant with the unweighted UniFrac kernel than with the CLR-linear kernel, we focus on unweighted UniFrac for our subsequent analysis of *MTMR12*. Figure 3 shows the Manhattan plots and linkage disequilibrium (LD) heatmaps from the second-stage variant-level analysis of the HCHS/SOL data, using the PC-adjusted KRV. The *IL23R* and *C1orf141* genes were combined into a single *IL23R-C1orf141* region due to overlapping variants. Based on the analysis using the Bray-Curtis kernel, there are 72 significant variants (out of 557 common variants) in the *IL23R-C1orf141* region ($\alpha = 0.05/557 = 8.98 \times 10^{-5}$). Based on the analysis using the unweighted UniFrac kernel, there are 114 significant variants (out of 288 common variants) in *ZFR* and 125 significant variants (out of 174 common variants) in *MTMR12* ($\alpha = 0.05/(288+174) = 1.08 \times 10^{-4}$). In addition, the Manhattan plot for *MTMR12* based on the CLR-linear kernel shows similar association patterns to the result based on unweighted UniFrac (Fig. S2). Relevant information including positions, rsID and *p*-values for these variants is reported in Table S3. From the LD heatmaps, in each gene, the significant variants share a high level of linkage disequilibrium with one another. Future fine mapping of causal variants that affect the microbiome composition will be needed.

**Fig. 4:**
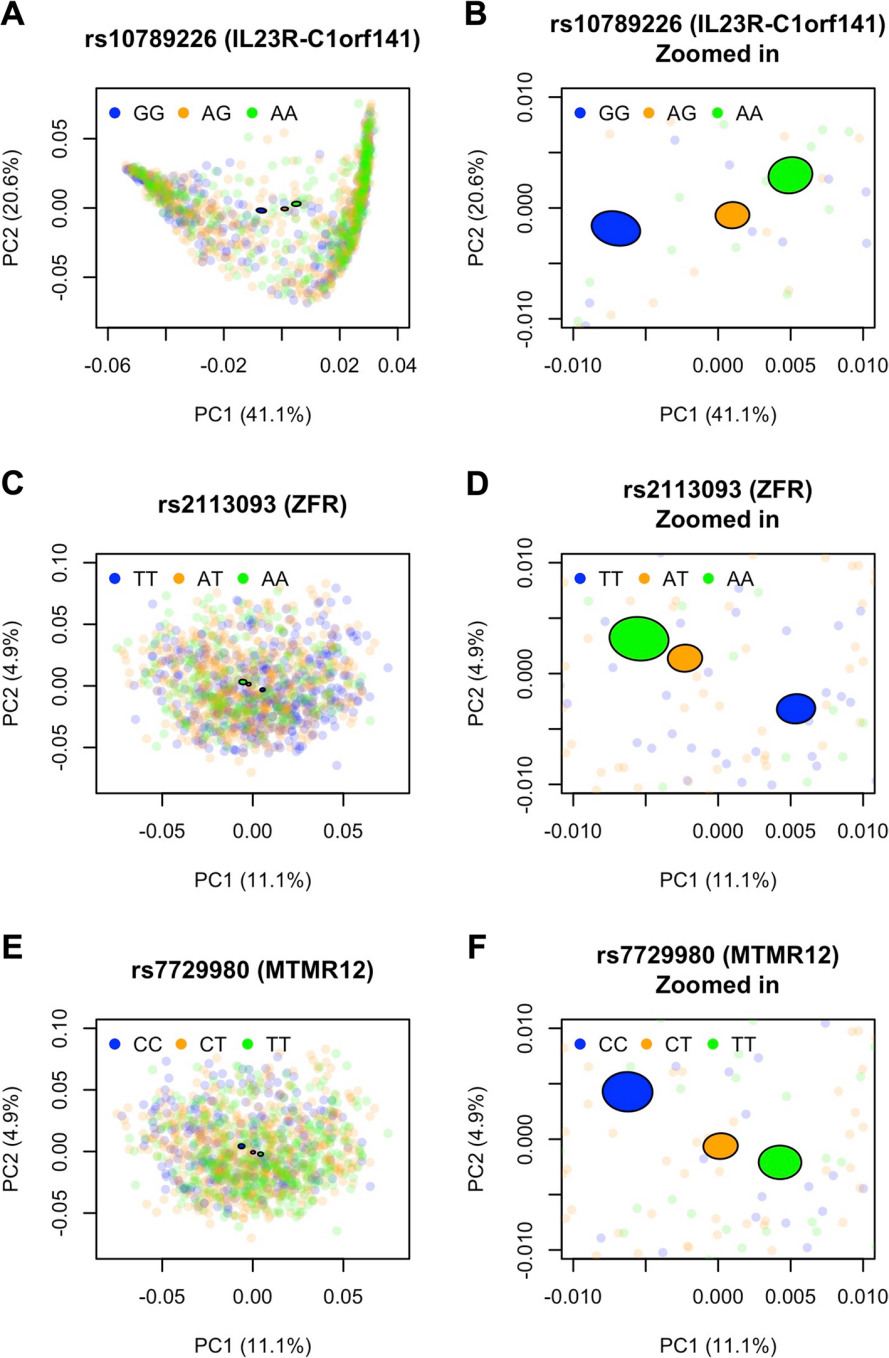
PC2 vs. PC1 from kernel PCA on the microbiome kernel, colored by genotype of top variants from the significant genes in the HCHS/SOL study. For each variant, a 95% confidence ellipse (shown as a filled ellipse with black borders) was constructed for individuals from each genotype. The Bray-Curtis kernel was used for the top variant in the *IL23R-C1orf141* region; the unweighted UniFrac kernel was used for the top variants in *ZFR* and *MTMR12*. The percent of variance captured by each kernel PC was provided in the axis labels. Panels **B**, **D**, and **F** show enlarged versions of the confidence ellipses from panels **A**, **C**, and **E**

To confirm the validity of the covariate-adjusted KRV approach, we conduct kernel PCA on the Bray-Curtis and unweighted UniFrac kernel matrices, and check whether individuals’ microbiome profiles, captured by the top two kernel PCs, differ by genotypes of the top (most significant) variant from each identified gene. This is similar to a PCoA analysis. Figure 4 shows that, for each top variant, the 95% confidence ellipses for different genotypes are well separated from one another, corroborating the findings by the adjusted KRV. Similar results are found for the CLR-linear kernel with respect to the top variant from *MTMR12* (Fig. S2).

### Specific taxa involved in microbiome GWAS associations

To further understand how the discovered genes drive differences in gut microbiome composition, we conduct an exploratory analysis to identify specific microbial taxa involved in the microbiome GWAS associations. Our strategy is to perform dimension reduction on both genetic and microbiome data and use correlation analyses to complement and help interpret our community-level analysis results.

The general analysis procedure is summarized in Fig. S3. As each gene-microbiome association signal appears to be driven by a single locus (as shown in the LD heatmaps from Fig. 3), we focus on the top variant from each identified gene for our analysis. On the other hand, we also use the leading 10 kernel PCs from each microbiome kernel to capture the major variation from the overall microbiome composition. For each gene-microbiome association, the specific variant and microbiome kernel used in the analysis are consistent with the association results in Table 1. In Step 1, among the top 10 microbiome kernel PCs, we identify kernel PCs that are significantly correlated with the top variant after adjusting for population structure (with false discovery rate (FDR) corrected *p*-value < 0.05 from linear regression): these kernel PCs represent the microbial community profiles that mainly drive the gene-microbiome associations. In Step 2, we inspect genus-level microbial abundance data and identify taxa that contribute the most to the significant kernel PCs from Step 1 (with absolute correlation between taxon abundance and kernel PC $\ge$ 0.5): these taxa dominate the microbial profiles captured by the kernel PCs and in turn drive the gene-microbiome associations.

The microbial taxa identified for each gene-microbiome association signal are listed in Table S4. Due to roles in immunity, we focus on findings related to *IL23R* and *ZFR* for a detailed discussion. We first discuss the taxa involved in the association between *IL23R* and the Bray-Curtis kernel. Allele A (vs. Allele G) of the top variant, rs10789226, from *IL23R* is positively associated with the abundance of *Bacteroides* and *Blautia*, while being negatively associated with the abundance of *Prevotella*. *Bacteroides* and *Prevotella* are the most abundant genera in this study (representing 23.7% and 25.0% abundances of all microbial taxa) and dominate the first PC of the Bray-Curtis kernel. These two genera have been studied extensively as metrics for dietary patterns [4, 27]. Interestingly, a higher *Prevotella*-to-*Bacteroides* ratio is associated with greater obesity in Hispanic/Latino populations based on a previous study using HCHS/SOL data [35]. In terms of relation to immunity disorders, a meta-analysis [72] suggests that patients with IBD are associated with a lower abundance of *Bacteroides* compared to healthy individuals, although mixed roles of *Bacteroides* have been reported in other studies [61]. On the other hand, while *Prevotella* species are classically considered as commensal bacteria, increased abundance of certain *Prevotella* strains has been associated with mucosal inflammation and linked to chronic inflammatory diseases [40]. Based on these findings, it appears that Allele A of rs10789226 might be associated with an overall healthier gut microbiome composition in Hispanic/Latino populations.

We next look at the taxa involved in the association between *ZFR* and the unweighted UniFrac kernel. Allele T (vs. Allele A) of the top variant, rs2113093, from *ZFR* is positively associated with the abundance of two unidentified genera from *Clostridiales* and *Ruminococcaceae*. As *Ruminococcaceae* is an order that belongs to the *Clostridiales* family, this result is consistent with the strength of the unweighted UniFrac kernel in utilizing phylogenetic information. *Ruminococcaceae* helps maintain the gut health by producing short-chain fatty acids (SCFAs) [28], and a decreased abundance of *Ruminococcaceae* has been associated with IBD disorders [71] and inflammation in hepatic encephalopathy [5]. On the other hand, several commensal *Clostridiales* strains have been shown to mediate effective immune response against colorectal cancer in mouse models [49]. These findings support the potential roles of *Clostridiales* and *Ruminococcaceae* bacteria in mediating the effect of *ZFR* in regulating innate immune response, and Allele T of rs2113093 is likely associated with a more favorable gut microbiome composition.

Overall, the above findings offer us a better understanding of the identified community-level associations. Nevertheless, due to heterogeneity in functions of individual bacterial species and strains, a higher study resolution will be required to further elucidate the mechanisms underlying the association between the identified genes and the gut microbiome.

### Comparison to competing methods and previous studies

As a comparison to our proposed covariate-adjusted KRV approach, we applied additional competing methods of microbiome GWAS to the same set of HCHS/SOL data ($n=1219$). We first performed two gene-based community-level analyses that rely on univariate microbiome phenotypes (i.e., only using the top kernel PC of the microbiome kernel matrix), denoted as linear regression and SKAT. Neither of the methods has identified any genome-widely significant signals (Manhattan plots in Figs. S4 and S5). Therefore, compared to univariate methods that identify the same type of genetic features (i.e., genes associated with the overall microbiome composition), our proposed KRV framework has a superior power in detecting associations.

We also performed a traditional variant-based taxon-level analysis to identify individual genetic variants associated with individual microbial genera. 89 relatively common genera (present in $\ge$ 10% of all individuals) were tested in the analysis.

At a study-wide significance level ($\alpha = 5 \times 10^{-8} / 89 = 5.6 \times 10^{-10}$), we have identified two associations that involve two genetic loci. The first association signal is between a block of $\sim$1 Mb region located at Chromosome 2 q21.3–q22.1, including 58 significant variants, and the abundance of *Bifidobacterium*. This locus involves the *LCT* gene and 8 other genes, exhibiting high-level LD among the significant variants. The top variant from this locus is rs4988235 (*p*-value = $4.2 \times 10^{-17}$), a functional variant associated with lactase persistence [21]. This signal was also reported by Kurilshikov et al. [38], who analyzed a sample of 18,340 individuals which consisted of 24 multi-ancestry cohorts including the HCHS/SOL GOLD cohort. In our gene-level analysis using the PC-adjusted KRV, the *LCT* gene is nominally significant based on the unweighted UniFrac kernel (*p*-value = 0.013), the CLR-linear kernel (*p*-value = 0.027) and the PhILR-linear kernel (*p*-value = 0.015), but not significant at the genome-wide level.

The second association signal is between a locus at Chromosome 18 q11.2, including 2 significant variants, and the presence/absence of *Christensenella* (top variant: rs1607482; *p*-value = $2.2 \times 10^{-10}$). This locus is intergenic, located between two RNA genes, *LINC01908* and *LOC105372038*. As our proposed analysis approach focused on gene regions only, these variants were not covered in our community-level analysis.

We next investigate the replication of signals found by previous gut microbiome GWAS studies in our analysis. We have examined the significance of 63 previously reported genes that harbor variants associated with gut microbiome beta-diversity [25, 43, 53, 62, 64] (Table S5). 59 out of 63 genes include at least one common variant in the HCHS/SOL data. Five genes are replicated with nominal significance (*p*-value < 0.05) based on various microbiome kernels: *BANK1* (unweighted UniFrac, weighted UniFrac), *MAST3* (weighted UniFrac, generalized UniFrac), *POMC* (CLR-linear), *C1orf21* (CLR-linear) and *AHSA2* (PhILR-linear). Among these genes, *POMC* produces peptides involved in anti-inflammatory actions [7], *BANK1* is associated with systemic lupus erythematosus [37], and *MAST3* and *AHSA2* are associated with IBD [39, 66], corroborating the role of immunity-related genes in shaping gut microbiota. However, none of the genes are significant at the genome-wide level.

### Simulation results

We have conducted simulation studies to further evaluate the performance of our proposed covariate-adjusted KRV test in terms of type I error rate and power. Table 2 shows the empirical type I error rates of both unadjusted and adjusted KRV tests at different significance levels under Type I Error Scenario 1. The unadjusted KRV has inflated type I error rates for all microbiome kernels except unweighted UniFrac. In contrast, the adjusted KRV maintains valid type I error rates for all microbiome kernels. Note that for Type I Error Scenario 1, population structure affected the abundance of common OTUs, which was unlikely to change these OTUs’ presence. Since the unweighted UniFrac kernel only captures presence/absence, but not abundance information of a taxon, the population stratification of microbiome profiles is not reflected in the unweighted UniFrac kernel. This absence of confounding effect leads to a valid type I error rate for the unweighted UniFrac kernel even when the unadjusted KRV is used.

Under Type I Error Scenario 2 (Table S2), where population structure affected the abundance of rare OTUs, the unadjusted KRV has highly inflated type I error rates for all microbiome kernels. Again, the adjusted KRV is able to maintain valid type I error rates for all microbiome kernels.

**Table 2:** Empirical type I error rate of unadjusted and covariate-adjusted KRV at nominal level $\alpha$ under Type I Error Scenario 1

Method	Microbiome kernel	$\alpha$		
		0.05	0.01	0.001
Unadjusted KRV	Bray-Curtis	0.2403	0.0936	0.0255
	Unweighted UniFrac	0.0484	0.0094	0.0011
	Weighted UniFrac	0.1371	0.0371	0.0057
	Generalized UniFrac	0.1412	0.0416	0.0063
	CLR-linear	0.0811	0.0178	0.0016
	PhILR-linear	0.1389	0.0434	0.0076
Adjusted KRV	Bray-Curtis	0.0473	0.0114	0.0012
	Unweighted UniFrac	0.0523	0.0115	0.0009
	Weighted UniFrac	0.0507	0.0095	0.0012
	Generalized UniFrac	0.0499	0.0097	0.0011
	CLR-linear	0.0450	0.0091	0.0011
	PhILR-linear	0.0482	0.0093	0.0015

Linear kernel was used for genetic data

**Fig. 5:**
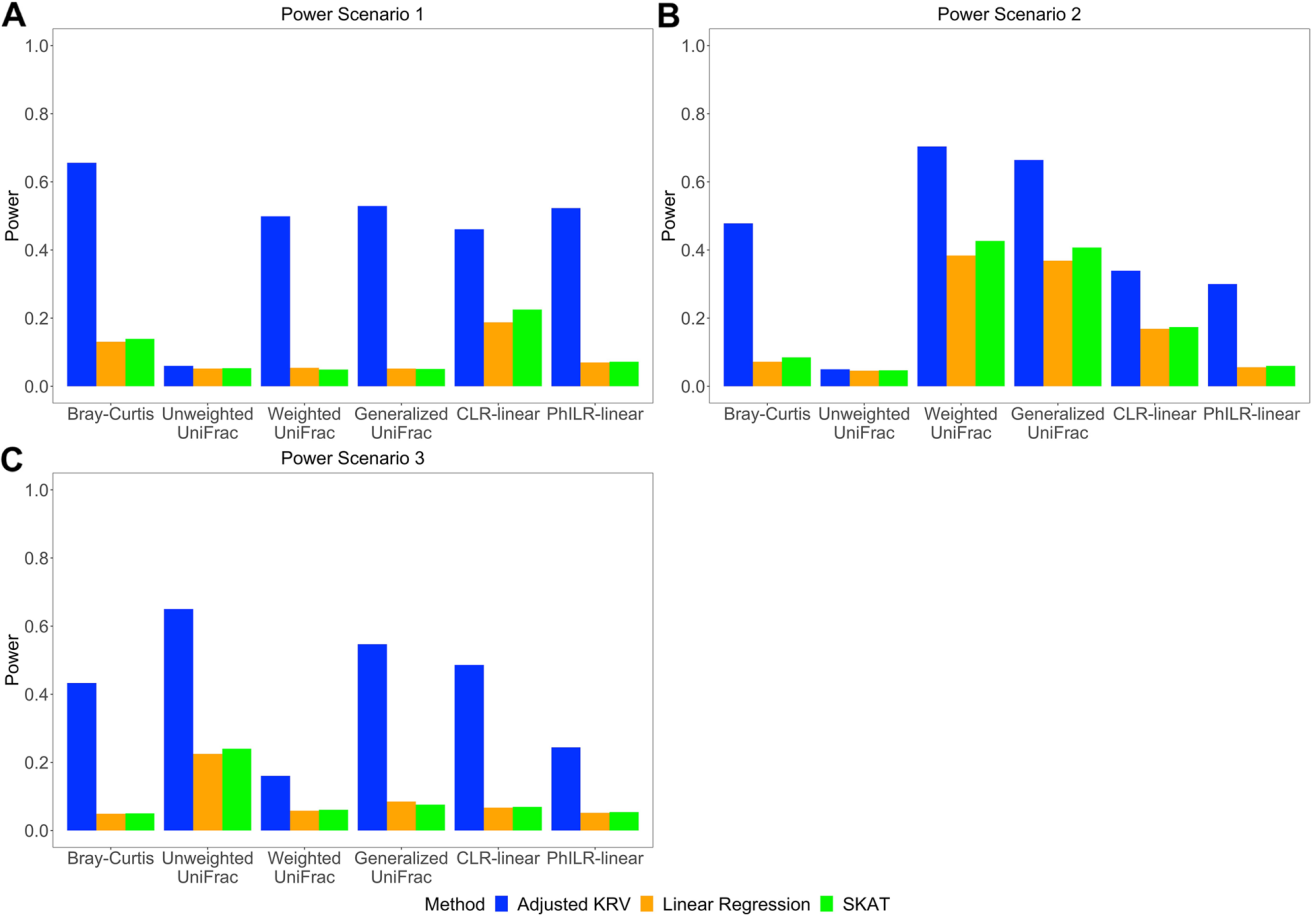
Empirical power of covariate-adjusted KRV and competing methods at nominal level $\alpha =0.05$ for different microbiome kernels under small effect sizes. **A** A single SNP affects the abundance of common OTUs. **B** A single SNP affects the abundance of OTUs from a common phylogenetic cluster. **C** A single SNP affects the abundance of rare OTUs. In each scenario, linear kernel was used for genetic data

Figure 5 shows the empirical power of the covariate-adjusted KRV test and competing methods under small effect sizes, at the nominal level $\alpha = 0.05$. In general, for each power scenario, the adjusted KRV has a much higher power than linear regression and SKAT, regardless of the microbiome kernel being used (with the exception of unweighted UniFrac in Power Scenario 1 and 2). Next we focus on the adjusted KRV and compare across microbiome kernels: in Power Scenario 1, the Bray-Curtis kernel has the highest power; in Power Scenario 2, the weighted UniFrac kernel has the highest power; in Power Scenario 3, the unweighted UniFrac kernel has the highest power. These results are consistent with the ways these microbiome similarity measures are constructed and can serve as clues as to which microbial features are affected when we use these kernels to detect associations in practice. The Bray-Curtis kernel is efficient in detecting abundance changes in common OTUs. The weighted UniFrac kernel has more power to detect abundance changes in common phylogenetic clusters, and the unweighted UniFrac kernel is more efficient in detecting changes in rare lineages. Again, due to the nature of unweighted UniFrac, all three methods based on this kernel have little power in Power Scenario 1 and 2, where the SNP effect on common OTUs or common phylogenetic clusters is unlikely to change their presence.

Under large effect sizes (Fig. S6), while the covariate-adjusted KRV displays a clear improvement in power, the overall patterns are similar to those under small effect sizes and again highlight the power gain of our proposed approach over univariate-phenotype-based competing methods.

## Discussion

Given the importance of the microbiome in human health, there is an emerging interest in studying the relationship between host genetic variation and human microbiome. Our methodological contribution in this work is twofold. First, we have proposed a novel microbiome GWAS approach to evaluate the association between gene-level genetic variation and community-level microbiome composition. Second, we have proposed a novel multivariate statistic, the covariate-adjusted KRV, to implement this approach with flexible covariate adjustment. By reducing the multiple-testing burden and aggregating small effect sizes between the genetics and the microbiome, our proposed approach improves statistical power and thus requires fewer samples to detect associations compared to the traditional marginal testing approach. Simulation studies show that the covariate-adjusted KRV maintains valid type I error rates in the presence of confounders and has a much higher power compared to other microbiome GWAS methods that rely on univariate microbiome phenotypes. In a genome-wide analysis of the HCHS/SOL data, we have identified four genes associated with gut microbiome beta-diversity. We have also identified individual variants within these genes and specific microbial taxa involved in the associations, which will be useful for future investigation of the mechanisms underlying the genetics-microbiome relationships.

Most of the identified genes based on the HCHS/SOL data have been previously implicated in immune functions or immunity-related disorders. This is consistent with the works by Blekhman et al. [6] and Rühlemann et al. [53], where loci in immunity-related genes and pathways have been shown to correlate with gut microbiome composition. The *IL23R* gene is especially interesting for future study, due to its recognition in previous microbiome genetic association studies [67] and its role in IBD, a chronic inflammatory disease that involves both genetic and microbial factors. Many genetic markers associated with IBD are involved in the interactions between the immune system and the microbiome [14, 34]. Furthermore, IBD is characterized by shift in the gut microbiome composition [36, 50], and specific microbes have also been shown to predict response to therapy [3] and postoperative disease recurrence [59] in patients with IBD. Therefore, our finding supports previous work and could contribute to future investigation of the disease etiology. Finally, as HCHS/SOL is one of the most comprehensive studies of Hispanic/Latino populations in the USA, the results from our analysis will help inform important genetic risk factors for gut-microbiome-related health outcomes in Hispanic/Latino individuals.

Although the covariate-adjusted KRV has valid type I error rates regardless of the kernels used, selecting appropriate kernels that reflect the actual patterns of association is important for maintaining a good statistical power. Different kernels measure different aspects of the structure within the data and assume different association patterns. For example, as we see from previous studies [70] and our simulations results, the Bray-Curtis kernel is more powerful in detecting associations where genetic variation affects common microbial taxa, whereas the unweighted UniFrac kernel is more powerful when genetics affects rarer phylogenetic clusters. In the analysis of the HCHS/SOL data, using different microbiome kernels, we discovered distinct significant genes. This is likely because these genes affect different aspects of the microbiome composition. For example, variants in the *IL23R-C1orf141* region, identified using Bray-Curtis, mainly associate with abundances of *Bacteroides* and *Prevotella* (Table S4), which are the most abundant genera in this data set. Variants in *ZFR* and *MTMR12*, identified using unweighted UniFrac, associate with genera from less abundant microbial lineages such as *Clostridiales* and *Ruminococcaceae* (Table S4). Often, we do not have prior knowledge on the ways genetics is associated with the microbiome. A possible extension would be to use an omnibus test that accommodates multiple possible kernels. For example, as proposed by Zhan et al. [68], we could construct an omnibus kernel matrix via a weighted sum of multiple candidate kernel matrices. Another approach would be to combine *p*-values obtained using different candidate kernels into a single *p*-value, such as the Cauchy *p*-value combination method [44].

While we mainly adjusted for population structure, a major confounder in the genetics-microbiome relationship, in our analysis of the HCHS/SOL data, adjusting for additional covariates (age, gender, and study sites) in a reduced sample revealed similar results. However, the signal from the *IL23R-C1orf141* region based on the Bray-Curtis kernel no longer has genome-wide significance in the latter analysis, which is a limitation of our study. Further analyses (Additional File 1: Section S3) suggest that this loss of power is likely due to sample size loss, rather than additional confounding or systematic differences from sub-sampling. Previous studies have reported that Bray-Curtis dissimilarity is less stable to sub-setting and aggregation of data than other types of dissimilarity/distance measures [24], which might also contribute to this reduced significance.

We have compared our gene-based community-level analysis to a traditional variant-based taxon-level microbiome GWAS conducted on the same data. While we identified an association between the *LCT* locus and *Bifidobacterium* abundance at a study-wide significance in the taxon-level analysis, the *LCT* gene was not genome-widely significant in the community-level analysis. *Bifidobacterium* was a relatively common genus (representing 1.04% abundance of all microbial genera) in the HCHS/SOL data. However, when we analyzed the microbiome as a whole and used microbiome kernels that are efficient in detecting abundance changes in common taxa, such as Bray-Curtis and weighted UniFrac, abundance differences in *Bifidobacterium* were likely overshadowed by those in the most abundant genera such as *Bacteroides* and *Prevotella*. This discrepancy in results reflects the inherent difference between taxon-level and community-level analyses. On the other hand, none of the genes identified in our community-level analysis was replicated in the taxon-level analysis, highlighting the value of our proposed approach in discovering gene-microbiome associations that involve concerted shifts in the microbial community. Nevertheless, our proposed KRV framework is not meant to replace the existing taxon-level microbiome GWAS approaches, as the two modes of analysis focus on distinct types of genetic features. If one is interested in identifying both loci associated with individual taxa and loci associated with the overall microbiome composition, our proposed framework can be applied in conjunction with existing taxon-level GWAS approaches to provide comprehensive results.

We have also investigated the replication of signals from previous gut microbiome GWAS studies. Five previously reported beta-diversity-associated genes [62] have been replicated in our analyses at a nominal significance, but none of the previous signals [25, 43, 53, 62, 64] reaches genome-wide significance. There are several possible reasons. First, compared to environmental effect, most host genetic influences on gut microbiome composition have relatively small effect sizes [52]. The sample sizes of current microbiome GWAS studies, including our study, are still too small to achieve enough statistical power. Second, there is considerable variation across studies in the collection and processing of microbiome data, leading to difficulties in reproducibility. Lastly, certain genetics-microbiome associations might be specific to ancestry or populations. In addition, since we focused on genetic loci within or close to gene regions, we were unable to evaluate the significance of previously identified loci that fell in intergenic regions.

While we have focused on the application of our proposed approach to microbiome GWAS in this work, the covariate-adjusted KRV can also be applied to investigate the relationships among other types of multivariate omics data. For example, we can investigate microbiome-metabolome relationships by examining the association between microbiome composition and groups of host metabolites that belong to distinct metabolic pathways. Such an analysis was described in one of our previous works [42], where we used a similar multivariate testing strategy to identify metabolic pathways associated with the vaginal microbiome. The advantages of reduced multiple testing burden and better captured data structure in our proposed approach can be readily carried over to other types of omics data.

## Conclusions

We have proposed a promising approach, the covariate-adjusted KRV framework, to study the covariate-adjusted association between host genetic variation and community-level microbiome composition, which demonstrates good performances in both simulations and real data analysis. The genes and loci identified using our approach will help elucidate the complex interactions among host genetics, gut microbiome and host immune systems. With the increasing collection of various omics data and high-dimensional traits, we expect the covariate-adjusted KRV to bring more discoveries by taking advantage of the innate structure within the omics and phenotypic data.

## Supplementary information

**Additional file 1.** A PDF file that includes additional methods and results (Section S1-S3), supplemental figures (Fig. S1-S7) and short tables (Table S1-S2).

**Additional file 2.** An XLSX file that includes supplemental long tables (Table S3-S5).

## Data Availability

The HCHS/SOL data used in our study are deposited at the database of Genotypes and Phenotypes (dbGap; http://view.ncbi.nlm.nih.gov/dbgap) and Biologic Specimen and Data Repository Information Coordinating Center (BIOLINCC; https://biolincc.nhlbi.nih.gov). The genotype and covariates data are available at dbGap under accession codes: phs000880.v1.p1 and phs000810.v1.p1. The 16S rRNA gene sequences are deposited in QIITA (https://qiita.ucsd.edu) under ID 11666, and European Nucleotide Archive (ENA; https://www.ebi.ac.uk/ena) under accession code ERP117287. HCHS/SOL has established a procedure for the scientific community to apply for access to participant data, with such requests reviewed by the Steering Committee of the HCHS/SOL project. These policies are described at https://sites.cscc.unc.edu/hchs. The covariate-adjusted KRV approach is implemented as part of the KRV() function in the MiRKAT R package v1.2.1, available at the Comprehensive R Archive Network (CRAN): https://cran.r-project.org/web/packages/MiRKAT. Instructions for usage and codes for reproduction of simulation results in this study are available at https://github.com/pearl-liu/Covariate-Adjusted-KRV. Figure 3 was produced using the LDheatmap R package v1.0: https://cran.r-project.org/web/packages/LDheatmap. The 95% confidence ellipses in Fig. 4 were produced using the ordiellipse() function of the vegan R package v2.5: https://cran.r-project.org/web/packages/vegan. Other tools include: *cosi2* program: https://software.broadinstitute.org/mpg/cosi2. SKAT R package v2.0.1: https://cran.r-project.org/web/packages/SKAT. GUniFrac R package v1.2: https://cran.r-project.org/web/packages/GUniFrac.
