# Supplementary material for: Kernel-based genetic association analysis for microbiome phenotypes identifies host genetic drivers of beta-diversity
Source: Microbiome. 2023 Apr 20;11:80. doi: 10.1186/s40168-023-01530-0 (PMC10116795; doi:10.1186/s40168-023-01530-0)
Supplement: Supplementary file 2 — Additional file 1. A PDF file that includes additional methods and results (Section S1-S3), supplemental figures (Fig. S1-S7) and short tables (Table S1-S2). [file 40168_2023_1530_MOESM1_ESM.pdf]

Supplementary Material for:

Kernel-based genetic association analysis for microbiome phenotypes identifies host genetic drivers of beta-diversity

Hongjiao Liu,[1,2] Wodan Ling,[3] Xing Hua,[2] Jee-Young Moon,[4]

Jessica S. Williams-Nguyen,[5] Xiang Zhan,[6] Anna M. Plantinga,[7] Ni Zhao,[8]

Angela Zhang,[1,2] Rob Knight,[9] Qibin Qi,[4] Robert D. Burk,[4,10]

Robert C. Kaplan,[2,4] and Michael C. Wu[1,2,*]

[1]Department of Biostatistics, University of Washington, Seattle, WA 98195, USA

[2]Public Health Sciences Division, Fred Hutchinson Cancer Center, Seattle, WA 98109, USA

[3]Division of Biostatistics, Department of Population Health Sciences, Weill Cornell Medicine, New York, NY 10065, USA

[4]Department of Epidemiology and Population Health, Albert Einstein College of Medicine, Bronx, NY 10461, USA

[5]Institute for Research and Education to Advance Community Health, Washington State University, Seattle, WA 98101, USA

[6]Department of Biostatistics and Beijing International Center for Mathematical Research, Peking University, Beijing 100191, China

[7]Department of Mathematics and Statistics, Williams College, Williamstown, MA 01267, USA

[8]Department of Biostatistics, Johns Hopkins University, Baltimore, MD 21205, USA

[9]Departments of Pediatrics and Computer Science & Engineering, University of California, San Diego, La Jolla, CA 92093, USA

[10]Departments of Pediatrics; Microbiology & Immunology; and, Obstetrics, Gynecology & Women's Health, Albert Einstein College of Medicine, Bronx, NY 10461, USA

[*]Correspondence: mcwu@fredhutch.org

S1 Derivation of covariate-adjusted KRV coefficient

Suppose that we have a phenotype kernel matrix $\boldsymbol{L}$ and a full-rank covariates matrix $\boldsymbol{X}$ that includes a column of 1's. We first perform a kernel principal component analysis (kernel PCA; equivalent to an eigendecomposition) on the phenotype kernel matrix and obtain a matrix $\boldsymbol{\Phi}$ such that:

$$\boldsymbol{L} = \boldsymbol{\Phi}\boldsymbol{\Phi}^T.$$

Here each column of $\boldsymbol{\Phi}$ is a kernel principal component (kernel PC) of $\boldsymbol{L}$ and has the form $\sqrt{\lambda_r}\boldsymbol{\phi}_r$ for $r = 1, \cdots, n$, where λ_r is the rth eigenvalue of $\boldsymbol{L}$ and $\boldsymbol{\phi}_r$ is the corresponding eigenvector for λ_r. We can view $\boldsymbol{\Phi}$ as a finite sample basis for the space spanned by the phenotype kernel function $\ell(\cdot, \cdot)$.

We then regress out the covariates $\boldsymbol{X}$ from each kernel PC:

$$\hat{\boldsymbol{\epsilon}} := \boldsymbol{\Phi} - \boldsymbol{P}_X\boldsymbol{\Phi},$$

where $\boldsymbol{P}_X = \boldsymbol{X}(\boldsymbol{X}^T\boldsymbol{X})^{-1}\boldsymbol{X}^T$ is the projection matrix onto the column space of $\boldsymbol{X}$. Now $\hat{\boldsymbol{\epsilon}}$ represents a sample basis that is orthogonal to the covariates $\boldsymbol{X}$. We can construct a new phenotype kernel matrix from this residual basis: $\boldsymbol{L}^* := \hat{\boldsymbol{\epsilon}}\hat{\boldsymbol{\epsilon}}^T$. Note that $\boldsymbol{L}^*$ can be expressed in terms of $\boldsymbol{L}$:

$$\boldsymbol{L}^* = (\boldsymbol{I} - \boldsymbol{P}_X)\boldsymbol{\Phi}\boldsymbol{\Phi}^T(\boldsymbol{I} - \boldsymbol{P}_X) = (\boldsymbol{I} - \boldsymbol{P}_X)\boldsymbol{L}(\boldsymbol{I} - \boldsymbol{P}_X) = \boldsymbol{P}_X^{\perp}\boldsymbol{L}\boldsymbol{P}_X^{\perp},$$

where we let $\boldsymbol{P}_X^{\perp} := \boldsymbol{I} - \boldsymbol{P}_X$. Similar procedures can be performed on the genotype kernel matrix $\boldsymbol{K}$ to obtain the adjusted genotype kernel matrix $\boldsymbol{K}^* := \boldsymbol{P}_X^{\perp}\boldsymbol{K}\boldsymbol{P}_X^{\perp}$. Both $\boldsymbol{K}^*$ and $\boldsymbol{L}^*$ are column-centered, since the covariates matrix $\boldsymbol{X}$ includes a column of 1's, accounting for the intercept in a regression. We can then construct a KRV statistic from the adjusted

kernel matrices $\boldsymbol{K}^*$ and $\boldsymbol{L}^*$:

$$\mathrm{KRV}_{adj}(G, Y|X) = \frac{\mathrm{tr}(\boldsymbol{K}^*\boldsymbol{L}^*)}{\sqrt{\mathrm{tr}(\boldsymbol{K}^*\boldsymbol{K}^*)\,\mathrm{tr}(\boldsymbol{L}^*\boldsymbol{L}^*)}} = \frac{\mathrm{tr}(\boldsymbol{P}_X^\perp\boldsymbol{K}\boldsymbol{P}_X^\perp\boldsymbol{L})}{\sqrt{\mathrm{tr}(\boldsymbol{P}_X^\perp\boldsymbol{K}\boldsymbol{P}_X^\perp\boldsymbol{K})\,\mathrm{tr}(\boldsymbol{P}_X^\perp\boldsymbol{L}\boldsymbol{P}_X^\perp\boldsymbol{L})}}.$$

Such a strategy of covariate adjustment can be seen as a special case of conditional independence (or uncorrelatedness) testing in a kernel-based framework, as proposed by Zhang et al. and Strobl et al. [9, 5]. In the context of microbiome GWAS, we are testing the correlation between genetic variants and microbiome community profiles, while conditioning on the covariates.

Special case of the linear kernel

Suppose that we use a linear kernel $\ell(\boldsymbol{y}_i, \boldsymbol{y}_j) = \boldsymbol{y}_i^T\boldsymbol{y}_j$ for the phenotype data, where $\boldsymbol{y}_i = (y_{i1}, \cdots, y_{iq})^T$ is the set of q traits for individual i.

Let $\boldsymbol{Y}$ be the $n \times q$ matrix that stores the phenotype data for all n individuals. Then the resulting phenotype kernel matrix can be constructed as $\boldsymbol{L} = \boldsymbol{Y}\boldsymbol{Y}^T$. Note that we can rewrite the covariate-adjusted kernel matrix $\boldsymbol{L}^*$ as:

$$\boldsymbol{L}^* = \boldsymbol{P}_X^\perp\boldsymbol{L}\boldsymbol{P}_X^\perp = \boldsymbol{P}_X^\perp\boldsymbol{Y}\boldsymbol{Y}^T\boldsymbol{P}_X^\perp = (\boldsymbol{P}_X^\perp\boldsymbol{Y})(\boldsymbol{P}_X^\perp\boldsymbol{Y})^T.$$

Therefore, in the case of a linear kernel, our proposed approach for covariate adjustment is equivalent to the previously proposed residual-based approach [6, 2, 8], where we first regress out the covariates from each raw phenotype and then construct the phenotype kernel matrix using the resulting residuals.

Connection between Euclidean distance and linear kernel

When constructing a microbiome kernel matrix, we can often obtain the kernel matrix by transforming existing distance or dissimilarity matrices calculated based on microbiome data. For example, assuming that the original microbial abundance data matrix is $\boldsymbol{Y}$, we can obtain a "CLR-Euclidean" kernel matrix by first constructing the Euclidean distance matrix $\boldsymbol{D}$ based on the CLR-transformed abundance data $\text{CLR}(\boldsymbol{Y})$ and then transforming $\boldsymbol{D}$ into a kernel matrix $\boldsymbol{L}$ via:

$$\boldsymbol{L} = -\frac{1}{2}\Big(\boldsymbol{I} - \frac{\mathbf{1}\mathbf{1}^T}{n}\Big)\boldsymbol{D}^2\Big(\boldsymbol{I} - \frac{\mathbf{1}\mathbf{1}^T}{n}\Big),$$

where $\boldsymbol{D}^2$ is the element-wise square of $\boldsymbol{D}$.

Now we show that, taking Euclidean distances of data $\text{CLR}(\boldsymbol{Y})$ followed by kernel matrix transformation is equivalent to constructing a centered linear kernel matrix based on $\text{CLR}(\boldsymbol{Y})$. For convenience, we still use $\boldsymbol{y}_i$ to represent the CLR-transformed abundances for individual i.

Let d_{ij}^2 be the (i,j)-th entry of matrix $\boldsymbol{D}^2$. Then we have

$$d_{ij}^2 = (\boldsymbol{y}_i - \boldsymbol{y}_j)^T(\boldsymbol{y}_i - \boldsymbol{y}_j) = \boldsymbol{y}_i^T\boldsymbol{y}_i - 2\boldsymbol{y}_i^T\boldsymbol{y}_j + \boldsymbol{y}_j^T\boldsymbol{y}_j.$$

As $\boldsymbol{H} := \boldsymbol{I} - \frac{\mathbf{1}\mathbf{1}^T}{n}$ is a centering matrix, the (i,j)-th entry of matrix $\Big(\boldsymbol{I} - \frac{\mathbf{1}\mathbf{1}^T}{n}\Big)\boldsymbol{D}^2\Big(\boldsymbol{I} - \frac{\mathbf{1}\mathbf{1}^T}{n}\Big)$

becomes

$$
\begin{aligned}
\tilde{d}_{ij}^2 &= d_{ij}^2 - \frac{1}{n}\sum_{i=1}^{n} d_{ij}^2 - \frac{1}{n}\sum_{j=1}^{n} d_{ij}^2 + \frac{1}{n^2}\sum_{i=1}^{n}\sum_{j=1}^{n} d_{ij}^2 \\
&= -2\Big[\boldsymbol{y}_i^T\boldsymbol{y}_j - \frac{1}{n}\sum_{i=1}^{n}\boldsymbol{y}_i^T\boldsymbol{y}_j - \frac{1}{n}\sum_{j=1}^{n}\boldsymbol{y}_i^T\boldsymbol{y}_j + \frac{1}{n^2}\sum_{i=1}^{n}\sum_{j=1}^{n}\boldsymbol{y}_i^T\boldsymbol{y}_j\Big] \\
&\quad + \Big[\boldsymbol{y}_i^T\boldsymbol{y}_i + \boldsymbol{y}_j^T\boldsymbol{y}_j\Big] - \frac{1}{n}\sum_{i=1}^{n}\Big[\boldsymbol{y}_i^T\boldsymbol{y}_i + \boldsymbol{y}_j^T\boldsymbol{y}_j\Big] - \frac{1}{n}\sum_{j=1}^{n}\Big[\boldsymbol{y}_i^T\boldsymbol{y}_i + \boldsymbol{y}_j^T\boldsymbol{y}_j\Big] + \frac{1}{n^2}\sum_{i=1}^{n}\sum_{j=1}^{n}\Big[\boldsymbol{y}_i^T\boldsymbol{y}_i + \boldsymbol{y}_j^T\boldsymbol{y}_j\Big] \\
&= -2\Big[\boldsymbol{y}_i^T\boldsymbol{y}_j - \frac{1}{n}\sum_{i=1}^{n}\boldsymbol{y}_i^T\boldsymbol{y}_j - \frac{1}{n}\sum_{j=1}^{n}\boldsymbol{y}_i^T\boldsymbol{y}_j + \frac{1}{n^2}\sum_{i=1}^{n}\sum_{j=1}^{n}\boldsymbol{y}_i^T\boldsymbol{y}_j\Big].
\end{aligned}
$$

Therefore, the (i, j)-th entry of matrix $\boldsymbol{L}$ is

$$
(\boldsymbol{L})_{i,j} = -\frac{1}{2}\tilde{d}_{ij}^2 = \boldsymbol{y}_i^T\boldsymbol{y}_j - \frac{1}{n}\sum_{i=1}^{n}\boldsymbol{y}_i^T\boldsymbol{y}_j - \frac{1}{n}\sum_{j=1}^{n}\boldsymbol{y}_i^T\boldsymbol{y}_j + \frac{1}{n^2}\sum_{i=1}^{n}\sum_{j=1}^{n}\boldsymbol{y}_i^T\boldsymbol{y}_j.
$$

Consequently, the resulting kernel matrix $\boldsymbol{L}$ is a centered linear kernel matrix based on CLR($\boldsymbol{Y}$): $\boldsymbol{L} = \boldsymbol{H}\boldsymbol{L}_0\boldsymbol{H}$, where $(\boldsymbol{L}_0)_{i,j} = \boldsymbol{y}_i^T\boldsymbol{y}_j$ and $\boldsymbol{H} = \boldsymbol{I} - \frac{\boldsymbol{1}\boldsymbol{1}^T}{n}$.

In light of this result, we can view the CLR-Euclidean kernel as a centered linear kernel applied to the CLR-transformed microbiome data (or denote it as the CLR-linear kernel). Applying our proposed covariate adjustment approach to the CLR-Euclidean kernel matrix is thus equivalent to using the residual-based approach on the CLR-transformed data (i.e., regressing out the covariates from the CLR-transformed data and constructing a kernel matrix based on the residuals).

S2 Taxon-level microbiome GWAS of the HCHS/SOL study

As a comparison to our proposed gene-based community-level microbiome GWAS framework, we performed a traditional variant-based taxon-level microbiome GWAS based on the same set of HCHS/SOL data ($n = 1219$) used in our main analysis, where we tested the association between individual genetic variants and individual microbial genera.

For genetic data, we applied the same quality control criteria as for the community-level analysis and focused on common genetic variants with minor allele frequency (MAF) ≥ 0.05 along the genome (including both coding and non-coding regions). For microbiome data, we focused our analysis on relatively common genera that are present in $\geq 10\%$ of all 1219 individuals under analysis.

To conduct association testing, we performed either linear regression or logistic regression depending on the prevalence of the genera, based on analysis procedures in previous microbiome GWAS studies [3, 7, 4]. Specifically, for genera present in $\geq 90\%$ of individuals, we performed rank normal transformation on the rarefied abundance data to encourage normality and used linear regression to assess the association between each rank-normal-transformed microbial abundance and each genetic variant. For genera present in $\geq 10\%$ but $< 90\%$ of individuals, the presence/absence of each genus was used as the outcome and associated with each genetic variant via logistic regression. Similar to the community-level analysis, the top 5 PCs of genome-wide genetic variability were included in the regression models as covariates. The genome-wide association testing was conducted using the GENESIS R package v2.28.0: `https://bioconductor.org/packages/GENESIS`.

S3 Analyses to assess the robustness of the *IL23R-C1orf141* signal

Based on our main analysis of 1219 HCHS/SOL subjects, we have identified genome-widely significant associations between variants in *IL23R* and *C1orf141* and gut microbiome composition using the Bray-Curtis kernel (Table 1), where population structure, a major confounder captured by the top 5 PCs of genetic variability, was adjusted. However, these two associations no longer have genome-wide significance in a reduced sample ($n = 1096$) where additional covariates (age, gender and study sites) were available and adjusted. To assess the robustness of these two signals, we have conducted several additional analyses.

First, to investigate if there is additional confounding caused by age, gender and study site, we have assessed the association between our identified loci and these covariates in the reduced sample. *IL23R* and *C1orf141* were combined into a single *IL23R-C1orf141* region due to overlapping variants. We applied the SNP-set kernel association test (SKAT) [6] to assess the association between age/gender and common variants in the *IL23R-C1orf141* region, with a linear model for age and a logistic model for gender. Since there were no available SKAT models to accommodate study site as an outcome, which is a categorical variable with four levels, we used linear regression to regress the genotype of the top variant (rs10789226) in the *IL23R-C1orf141* region on study site. In all models, the population structure captured by the top 5 genome-wide genetic PCs were adjusted. We found no significant association between the genetics and any of the covariates (p-values for age, gender and study site were 0.06, 0.08 and 0.75, respectively), thus confirming that these covariates are not likely to be confounders in the genetics-microbiome relationship in our study.

Next, to discover any systematic differences between participants with ($n = 123$) and

without missing data ($n = 1096$) for the three covariates, we have compared the overall microbiome composition and genetic features of the *IL23R-C1orf141* region between these two sub-samples. We plotted the top two kernel PCs of the Bray-Curtis microbiome kernel matrix to identify any clustering by sub-samples and conducted permutational multivariate analysis of variance (PERMANOVA) [1] to test the difference between the two sub-samples in microbiome composition (see Figure S7). Similar analysis was performed for the genetic kernel matrix, constructed based on common variants in the *IL23R-C1orf141* region using a linear kernel. These analyses revealed no significant difference between participants with and without missing covariates data (PERMANOVA p-value = 0.07 for microbiome; 0.40 for genotypes).

Based on the above analyses, we have further confirmed that there is not likely to be systematic differences between the original sample and the reduced sample, and the additionally adjusted covariates are not likely to be confounders in the genetics-microbiome relationship. These results have confirmed the robustness of our identified genetic loci based on the Bray-Curtis kernel, and the reduced genome-wide significance in the reduced sample is likely due to sample size loss.

Supplementary tables and figures

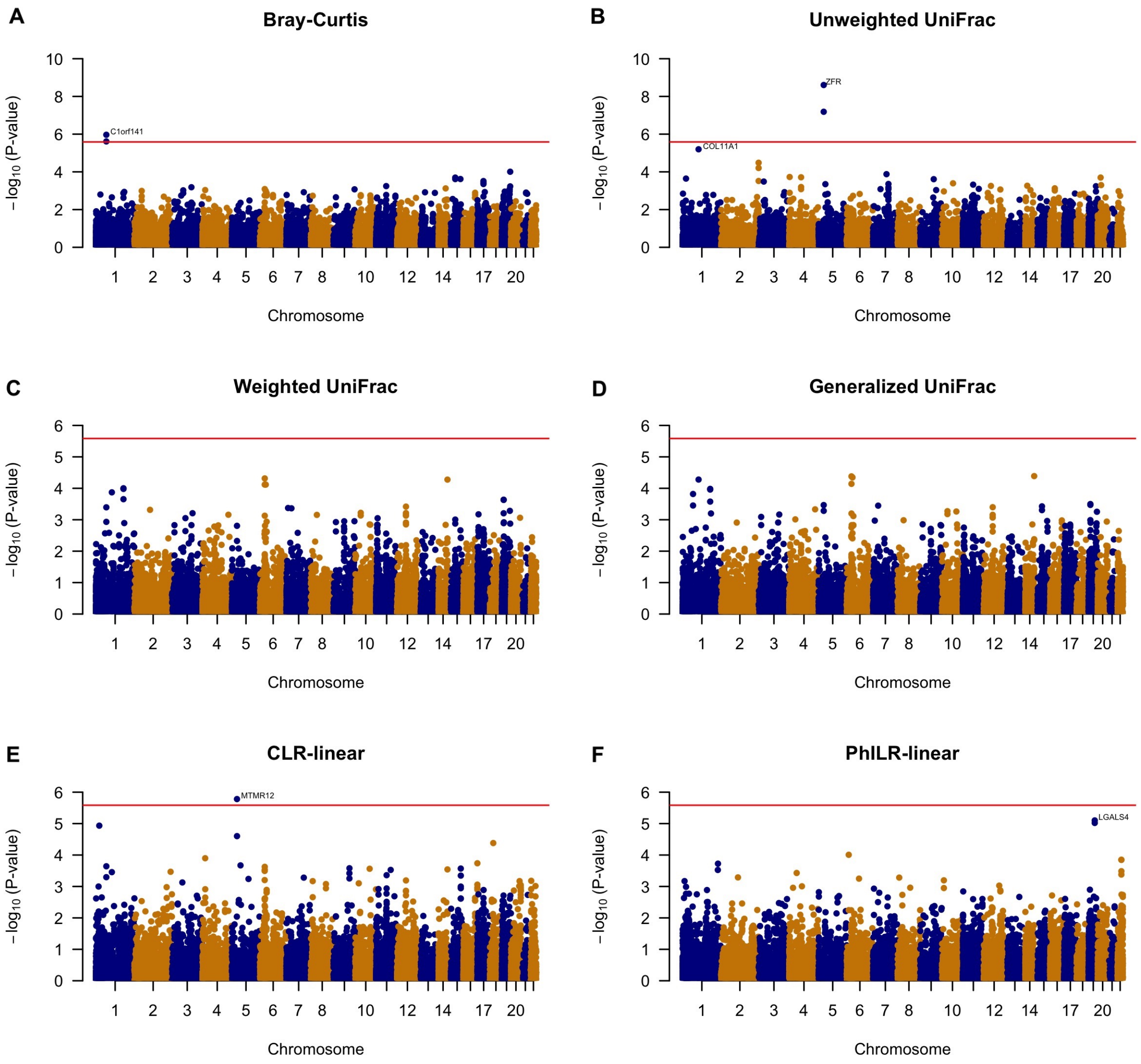

Figure S1: **Manhattan plots from the first-stage gene-level analysis of the HCHS/SOL data, using the PC-adjusted KRV.** Each panel corresponds to a distinct microbiome kernel. The top 5 PCs of genome-wide genetic variability were adjusted. The red lines represent the genome-wide significance threshold ($\alpha = 2.6 \times 10^{-6}$).

Table S1: P-values for the significant genes from Table 1 when additional covariates were adjusted in the first-stage KRV analysis of the HCHS/SOL data.

Microbiome kernel	Genes	Number of common variants	P-value
Bray-Curtis	*C1orf141*	484	2.5×10^{-5}
	IL23R	284	3.7×10^{-5}
Unweighted UniFrac	*MTMR12*	174	2.3×10^{-7}
	ZFR	288	3.3×10^{-8}
CLR-linear	*MTMR12*	174	3.3×10^{-6}

Adjusted covariates include the top 5 PCs of genome-wide genetic variability, age, gender and study sites. The analysis was performed on 1096 unrelated individuals where all relevant data were available.

Table S2: Empirical type I error rate of unadjusted and covariate-adjusted KRV at nominal level α under Type I Error Scenario 2.

Method	Microbiome kernel	α		
		0.05	0.01	0.001
Unadjusted KRV	Bray-Curtis	1.0000	1.0000	1.0000
	Unweighted UniFrac	1.0000	1.0000	1.0000
	Weighted UniFrac	0.9980	0.9794	0.8312
	Generalized UniFrac	1.0000	1.0000	1.0000
	CLR-linear	1.0000	1.0000	1.0000
	PhILR-linear	1.0000	1.0000	0.9983
Adjusted KRV	Bray-Curtis	0.0489	0.0104	0.0014
	Unweighted UniFrac	0.0473	0.0079	0.0007
	Weighted UniFrac	0.0482	0.0102	0.0018
	Generalized UniFrac	0.0467	0.0096	0.0009
	CLR-linear	0.0521	0.0116	0.0010
	PhILR-linear	0.0524	0.0094	0.0018

Linear kernel was used for genetic data.

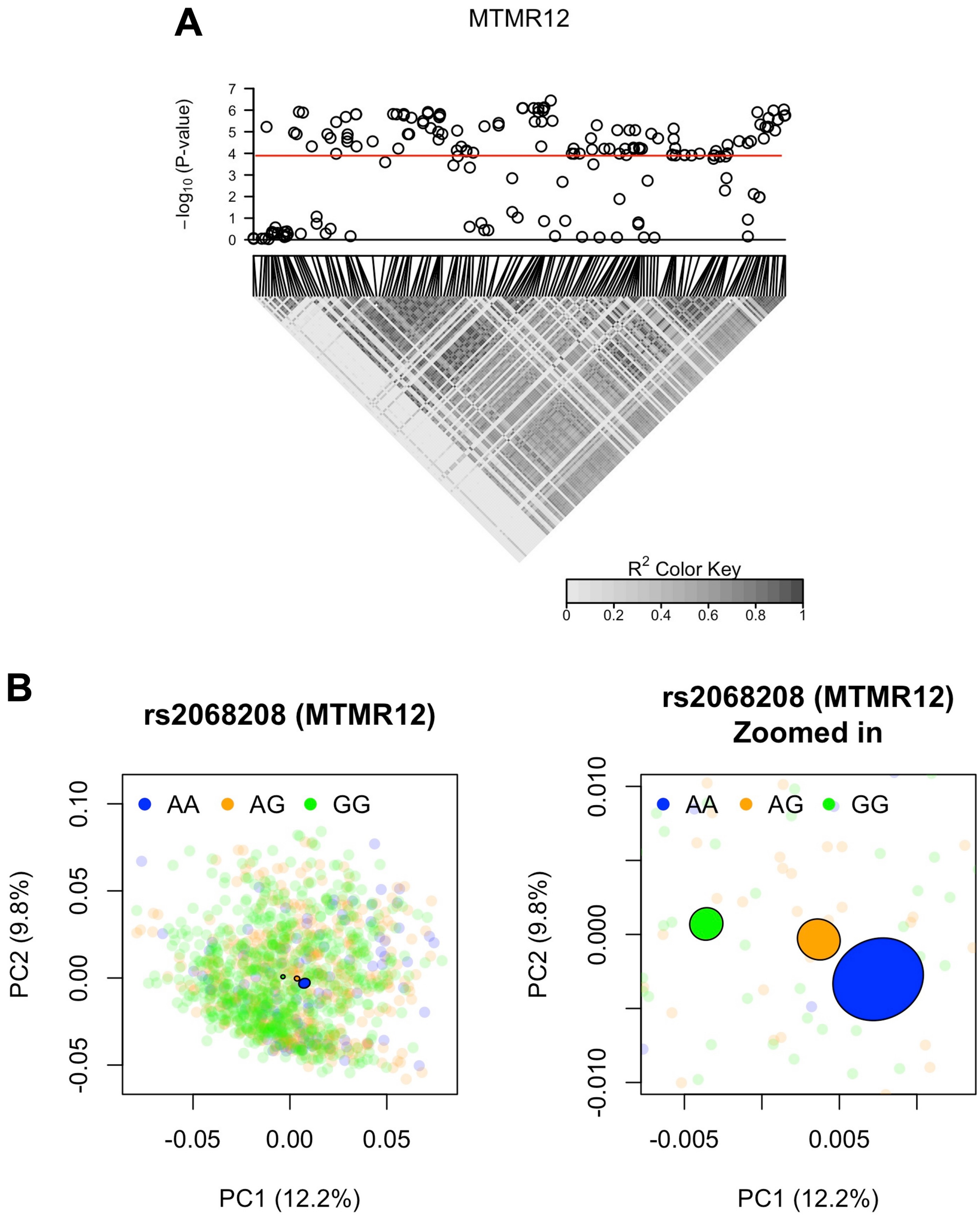

Figure S2: **Microbiome GWAS results of *MTMR12*, based on the CLR-linear kernel.** Panel (A): Manhattan plot and linkage disequilibrium (LD; R^2) heatmap from the second-stage variant-level analysis of the HCHS/SOL data, using the PC-adjusted KRV. The red line represents variant-level significance ($\alpha = 1.08 \times 10^{-4}$) used in the main analysis. Panel (B): PC2 vs. PC1 from kernel PCA on the CLR-linear kernel, colored by genotype of the top variant from *MTMR12*. The percent of variance captured by each kernel PC was provided in the axis labels.

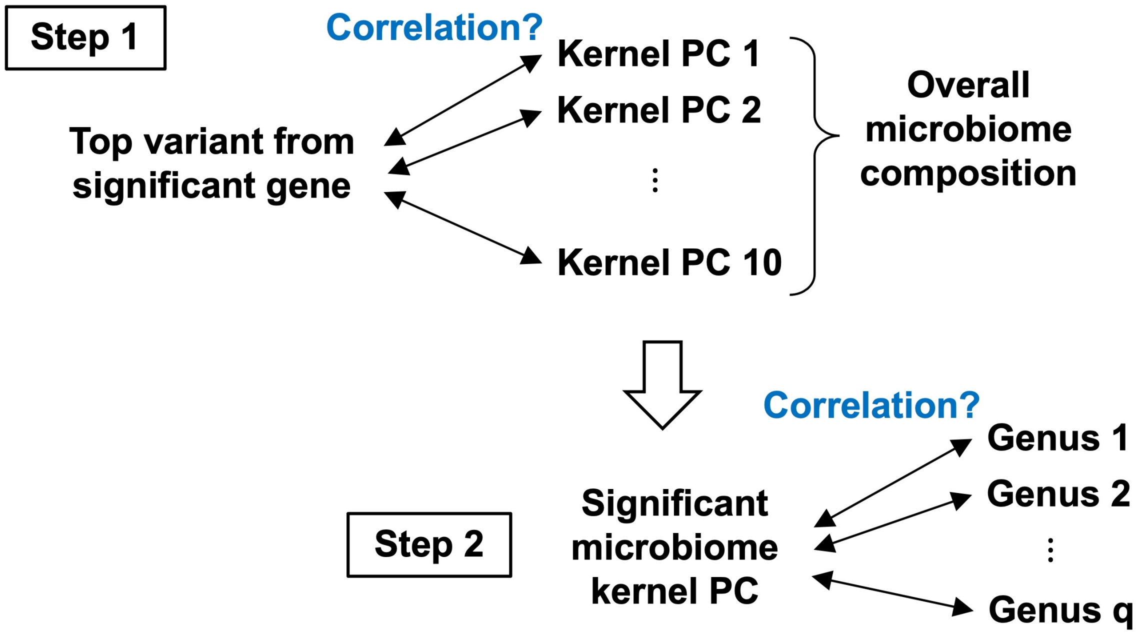

Figure S3: **Illustration of procedures to identify specific microbial taxa involved in the community-level microbiome GWAS associations.**

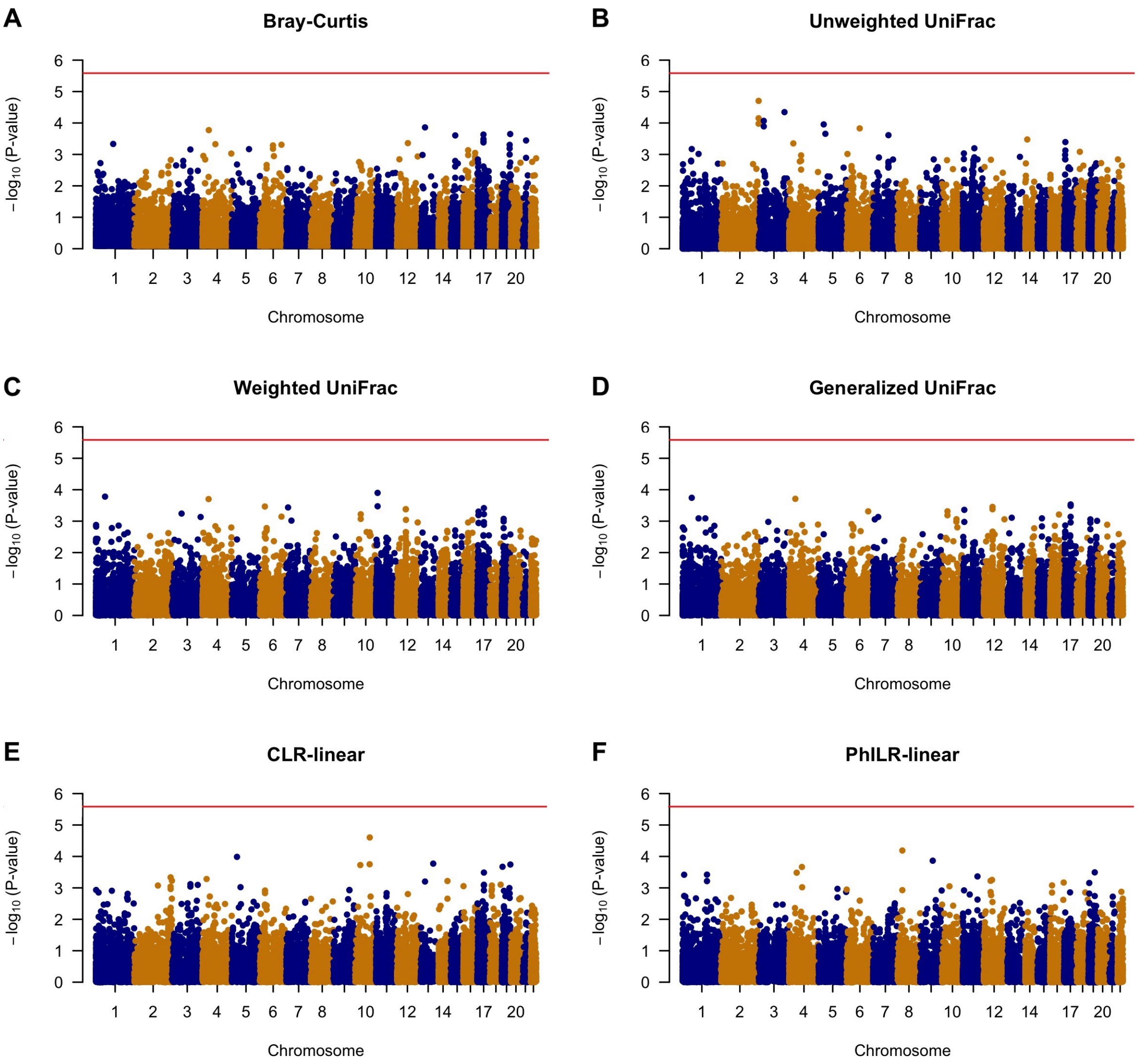

Figure S4: **Manhattan plots from alternative analysis of the HCHS/SOL data, via linear regression of the top PC of the community-level microbiome kernel matrix on the top PC of the gene-level genotype kernel matrix.** Each panel corresponds to a distinct microbiome kernel. The top 5 PCs of genome-wide genetic variability were adjusted. The red lines represent the genome-wide significance threshold ($\alpha = 2.6 \times 10^{-6}$).

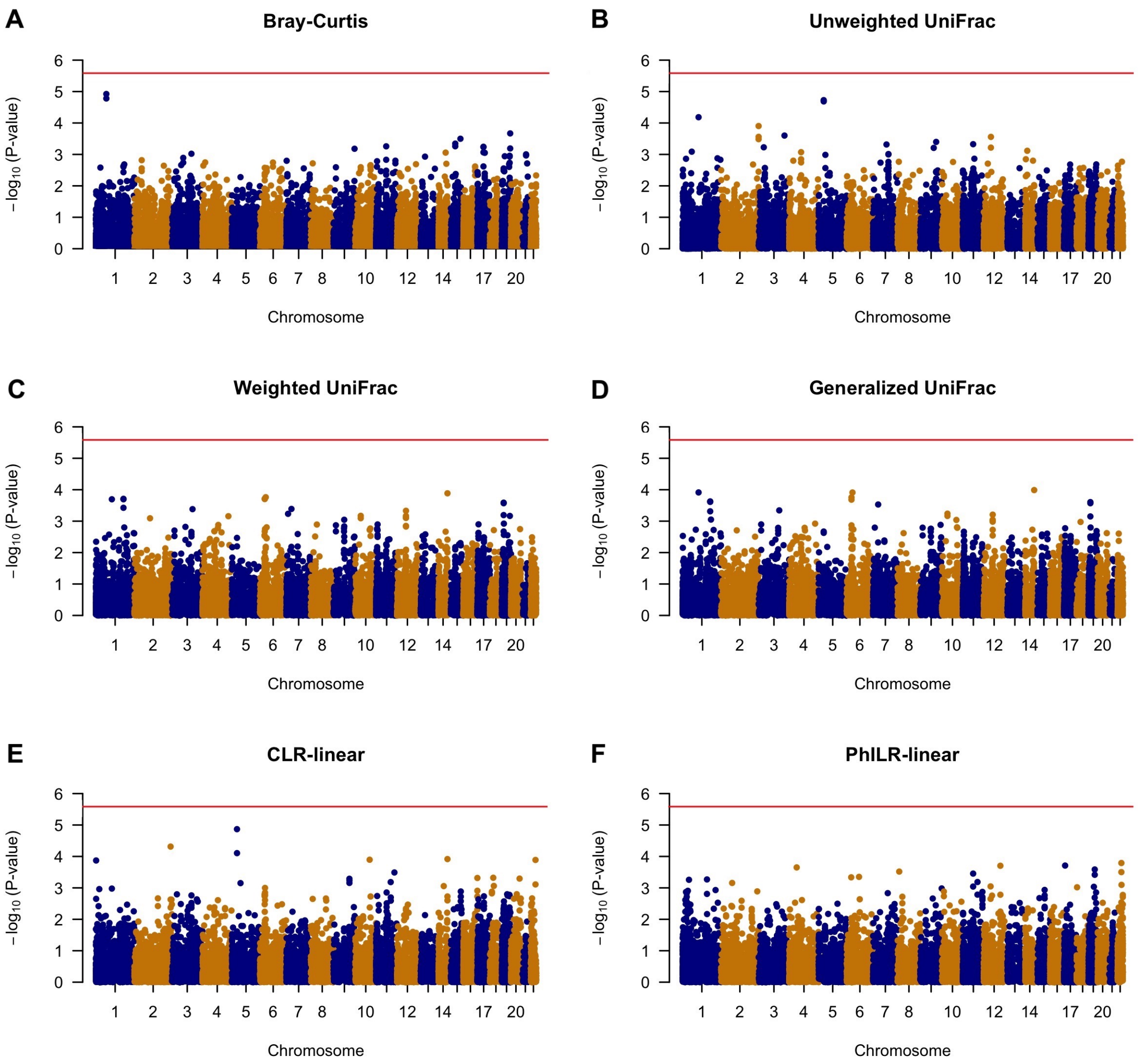

Figure S5: **Manhattan plots from alternative analysis of the HCHS/SOL data, via SKAT test of the top PC of the community-level microbiome kernel matrix on gene-level genetic variation.** Each panel corresponds to a distinct microbiome kernel. The top 5 PCs of genome-wide genetic variability were adjusted. The red lines represent the genome-wide significance threshold ($\alpha = 2.6 \times 10^{-6}$).

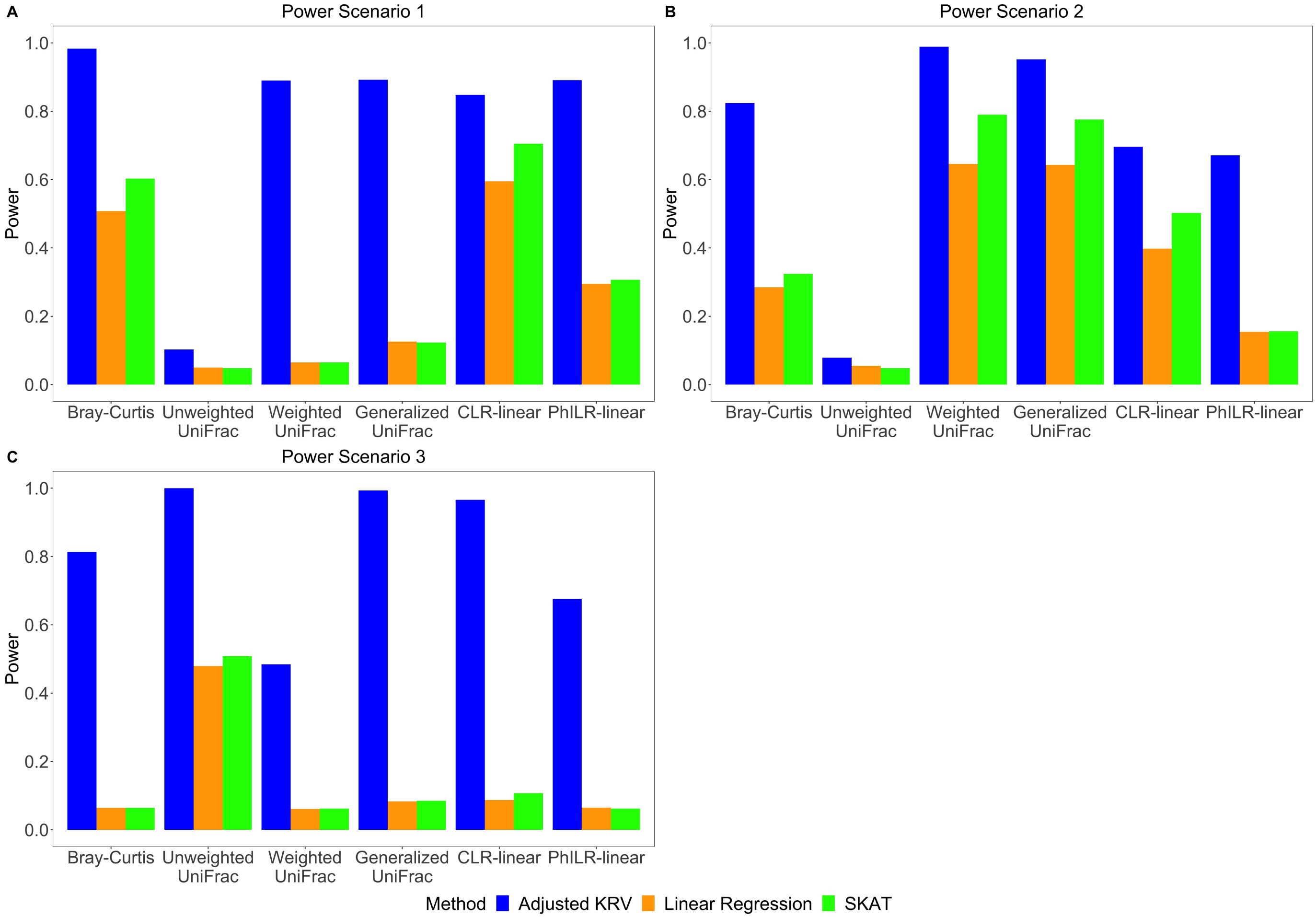

Figure S6: **Empirical power of covariate-adjusted KRV and competing methods at nominal level $\alpha = 0.05$ for different microbiome kernels under large effect sizes.** Panel (A): A single SNP affects the abundance of common OTUs. Panel (B): A single SNP affects the abundance of OTUs from a common phylogenetic cluster. Panel (C): A single SNP affects the abundance of rare OTUs. In each scenario, linear kernel was used for genetic data.

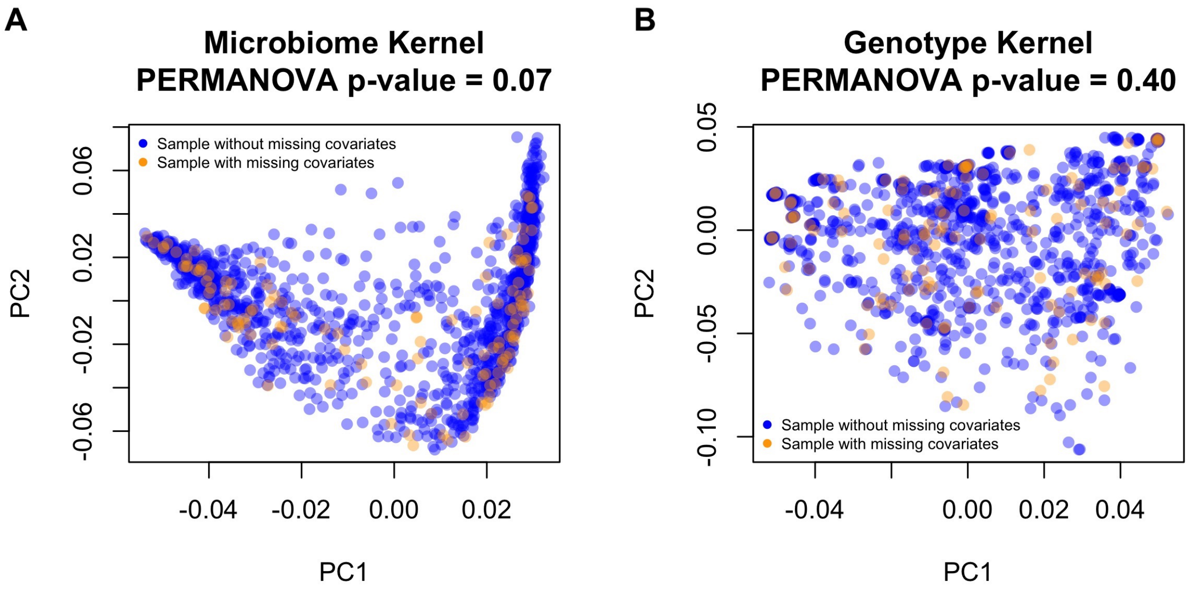

Figure S7: **PC2 vs. PC1 from kernel PCA on the Bray-Curtis microbiome kernel and the *IL23R-C1orf141* genotype kernel, colored by missing status of three covariates: age, gender and study site.** Panel (A): Kernel PCA was conducted on the Bray-Curtis microbiome kernel matrix. Panel (B): Kernel PCA was conducted on the linear genotype kernel matrix, which was constructed based on common variants in the *IL23R-C1orf141* region.

References

[1] Anderson, M. J. (2014). Permutational multivariate analysis of variance (PERMANOVA). *Wiley Statsref: Statistics Reference Online*, pages 1–15.

[2] Broadaway, K. A., Cutler, D. J., Duncan, R., Moore, J. L., Ware, E. B., Jhun, M. A., Bielak, L. F., Zhao, W., Smith, J. A., Peyser, P. A., et al. (2016). A statistical approach for testing cross-phenotype effects of rare variants. *The American Journal of Human Genetics*, 98(3):525–540.

[3] Hughes, D. A., Bacigalupe, R., Wang, J., Rühlemann, M. C., Tito, R. Y., Falony, G., Joossens, M., Vieira-Silva, S., Henckaerts, L., Rymenans, L., et al. (2020). Genome-wide associations of human gut microbiome variation and implications for causal inference analyses. *Nature Microbiology*, 5(9):1079–1087.

[4] Kurilshikov, A., Medina-Gomez, C., Bacigalupe, R., Radjabzadeh, D., Wang, J., Demirkan, A., Le Roy, C. I., Garay, J. A. R., Finnicum, C. T., Liu, X., et al. (2021). Large-scale association analyses identify host factors influencing human gut microbiome composition. *Nature Genetics*, 53(2):156–165.

[5] Strobl, E. V., Zhang, K., and Visweswaran, S. (2019). Approximate kernel-based conditional independence tests for fast non-parametric causal discovery. *Journal of Causal Inference*, 7(1).

[6] Wu, M. C., Lee, S., Cai, T., Li, Y., Boehnke, M., and Lin, X. (2011). Rare-variant association testing for sequencing data with the sequence kernel association test. *The American Journal of Human Genetics*, 89(1):82–93.

[7] Xu, F., Fu, Y., Sun, T.-y., Jiang, Z., Miao, Z., Shuai, M., Gou, W., Ling, C.-w., Yang, J., Wang, J., et al. (2020). The interplay between host genetics and the gut microbiome re-

veals common and distinct microbiome features for complex human diseases. *Microbiome*, 8(1):1–14.

[8] Zhan, X., Zhao, N., Plantinga, A., Thornton, T. A., Conneely, K. N., Epstein, M. P., and Wu, M. C. (2017). Powerful genetic association analysis for common or rare variants with high-dimensional structured traits. *Genetics*, 206(4):1779–1790.

[9] Zhang, K., Peters, J., Janzing, D., and Schölkopf, B. (2011). Kernel-based conditional independence test and application in causal discovery. In *Proceedings of the Twenty-Seventh Conference on Uncertainty in Artificial Intelligence*, UAI'11, page 804–813, Arlington, Virginia, USA. AUAI Press.
